# Polysubstance use patterns and novel synthetics: A cluster analysis from three U.S. cities

**DOI:** 10.1371/journal.pone.0225273

**Published:** 2019-12-03

**Authors:** Luther Elliott, Christopher Keith Haddock, Stephanie Campos, Ellen Benoit

**Affiliations:** 1 New York University, College of Global Public Health, Center for Drug Use and HIV/HCV Research, New York, New York, United States of America; 2 National Development and Research Institutes—USA, Leawood, Kansas, United States of America; 3 New York State Psychiatric Institute, Columbia University, New York, New York, United States of America; 4 North Jersey Community Research Initiative, Research Division, Newark, New Jersey, United States of America; University of California San Diego, UNITED STATES

## Abstract

The rapid emergence of novel psychoactive substances within the past decade has raised new concerns about the harms associated with unregulated drug use. Synthetic analogues—chemically related to established psychoactive substances like *cannabis sativa* and *catha edulis*—in particular have proliferated rapidly, allowing little opportunity for scientific research or the establishment of informal guidelines for safe use among consumers. To explore how synthetic substance use relates to other forms of use, this paper presents an analysis of polysubstance use among a sample of 676 people who use illicit substances in the United States. Participants were sampled from three greater metropolitan areas (Houston/Galveston, Texas; New York City; and New Orleans, Louisiana). Study researchers used cluster-type analyses to develop dendrogram visualizations of the interrelationships between substance types. Results suggest a considerable variation in substance and polysubstance use patterns across states in the U.S. Polysubstance use clustered around well-observed combinations like MDMA/cannabis and cocaine/heroin. Synthetic cannabinoids and cathinones showed no strong clustering with other substances. High rates of binge drinking among users of other substances further support the importance of interventions sensitive to the clinical challenges of polysubstance use.

## Introduction

The use of multiple substances within a specified period of time, or “polysubstance use,[1]” is a common practice among people who use illicit drugs or misuse prescribed drugs [1–3]. Polysubstance use has emerged as a particular topic in recent years, due in part to the rapid development and distribution of novel psychoactive substances (NPS)—typically chemical analogues of established drugs—and the high rate of opioid-involved polysubstance overdose deaths. Concurrent use of opioids with alcohol and other central nervous system depressants, such as benzodiazepine-class sedatives, are well known overdose risk factors [4], but many other less common drug combinations also present grave health risks to their users. For example, alcohol combined with gamma-hydroxybutyrate (GHB) presents serious risk of respiratory depression [5–8] and inadvertent combinations of monoamine oxidase inhibitors (MAOIs)—an essential ingredient in *ayahuasca* preparations—can lead to coma or even death among those prescribed selective serotonin reuptake inhibitors (SSRIs) for depression [9] or among those using ecstasy/MDMA recreationally [10, 11]. Some other common substance combinations, such as alcohol and cocaine, may impact concurrent users over time. Cocaethylene, a metabolite of cocaine formed by liver enzymes when alcohol is present [12, 13], appears acutely toxic in mice at lower concentrations than cocaine alone [14], suggesting the importance of ongoing study in humans.

In some contexts, the toxic side effects related to contraindicated polysubstance use are relatively well understood, such as among subcultures of club drug use, which have benefitted from harm reduction outreach and messaging distributed at raves, festivals, and nightclubs. However, the rapid emergence of novel psychoactive substances (NPS) in the past decade [15–18] has heightened concerns about the unanticipated consequences resulting from concomitant use of multiple substances. Analogues of cannabis’s most active cannabinoid, Tetrahydrocannabinol (THC), proliferated during a period of emergency scheduling by the FDA and DEA in the U.S., resulting in more than 130 currently identified variants [19], at least some of which have been linked to widespread overdoses and psychotic episodes [20–24]. As yet, however, no clinical work has examined the role of other concurrently-used substances in the precipitation of psychosis-like events following inhalation of synthetic cannabinoids sprayed on inert plant matter and sold as “spice” or “K2.”

The same concerns also hold for the wide array of chemical analogues of cathinone, commonly marketed and referred to as “bath salts” in the U.S. Synthetic cathinones—chemical analogues of psychoactive components in *Catha edulis*, or khat, a plant chewed for its stimulating properties throughout the Arabian Peninsula and East Africa [25–27]—vary considerably in reported effects. While the first cathinone analogues were synthesized as early as 1928 [28, 29], they remained relatively unknown to people who use illicit substances and the general public until reintroduced in U.S. gas stations, tobacconists, and “head shops” as shoe deodorizers, plant foods, or bath salts. Fueled by both increasing emergency department visits resulting from bath salt consumption—many involving cardiac events [30–32]—and a growing array of sensational stories regarding the dangerous consequences of cathinone use, the U.S. Congress introduced a series of synthetic drug control bills in 2011 explicitly targeting cannabis and cathinone analogues [33]. While consumers of synthetic cannabinoids and cathinones in most U.S. states can no longer purchase the substances legally, news media and emergency department reports suggest ongoing use [34, 35]. Given the relative lack of scientific knowledge about these emergent synthetic drugs, an epidemiological focus on how their use may cluster within patterns of polysubstance use remains timely.

Experiential reports of synthetic drug effects suggest some potential research questions. A number of cathinone variants are frequently likened to (meth)amphetamine in their effects while others share strong experiential similarities with 3,4-methyl enedioxy methamphetamine (MDMA), the club drug commonly referred to as ecstasy or molly in the U.S. (henceforth, “molly”). These perceived similarities have resulted in the common substitution of cathinones for MDMA in ecstasy tablets [36]. In the case of synthetic cannabinoids, individuals on probation or parole, as well as those with regular job-related urinalysis requirements, have often reported use of K2 or Spice as a cannabis replacement that has been, until recently, undetectable on standard urinalysis screens [37]. As a result, the present analysis was designed, in part, to investigate whether cathinone use clusters disproportionately among those who also use methamphetamine or molly and whether synthetic cannabinoid use was primarily unrelated to recent use of cannabis, potentially suggesting widespread substitution of a relatively high-risk alternative for traditional cannabis use. We also focus on how alcohol use and binge drinking may cluster with the use of illicit substances. Following the literature cited above, the additional euphoria linked to cocaethylene [38, 39] and observed subcultural patterns of concurrent alcohol and cocaine use among young club-goers [40, 41] and certain occupational groups [42, 43] could result in a disproportionate use of alcohol among people who use cocaine. Relationships between alcohol and synthetics have not been adequately explored in empirical literature, and our analyses that follow are therefore exploratory.

In presenting this preliminary analysis of polysubstance use patterns and the place of novel synthetic drugs therein, our work is informed by others in the field examining latent classes or clusters of polysubstance use [44–46]. Situating the work across three urban areas in the U.S., we have also aimed to provide some preliminary context about the potential for regional differences in synthetic and other substance use. While national household survey data indicate broad trends in major categories of illicit substance use by state, instrumentation has been slow to adopt measures of polysubstance or NPS use. Accordingly, important variances in such outcomes as opioid-involved overdose mortality between states like New York and Texas (16.1 per vs. 5.1 per 100,000 population, respectively)—despite both states having equivalent rates of past-year adult-age heroin use (.33% vs. .32%, respectively)—remain hard to interpret [47, 48]. While our work here is not designed or powered to be able to disentangle complex relationships between market dynamics and culturally and economically motivated use patterns, it is offered as a preliminary effort to better frame research questions about how distinct classes of polysubstance use may be critical drivers of health outcomes for people who use substances. Only through more contextually sensitive inquiry can research better achieve the goal of informing targeted forms of public health messaging, outreach, and intervention that might mitigate the negative health impacts of polysubstance use.

## Materials and methods

### Study overview

This analysis emerges from an NIH/NIDA-funded (R01DA035887) study of illicit substance use in 4 U.S. cities which was designed to yield findings about how cathinone/bath salt market structure, dynamics and related violence may differ from established drug markets for cocaine, methamphetamine and heroin.

#### Sampling approach

The mixed method study involved a quantitative survey data collected in Galveston and Houston, Texas; New York City; and New Orleans, Louisiana and reported on in this analysis as well as a qualitative substudy reported upon elsewhere [49]. In the course of earlier research in those cities, members of the study team learned about the appearance of synthetic cathinones and obtained funding to investigate the emerging market. The four sites were selected in part due to the advantages of having an existing team of skilled local field staff with extensive experience recruiting people who use drugs for earlier research [50–52].

The six study field staff conducted non-probabilistic sampling using a combination of peer chain referral and venue-based sampling to recruit 676 participants. The field staff, who resided in each target metropolitan area were advised to locate well-networked people who use drugs known from earlier studies where possible and also to start other participant referral chains by recruiting new participants approached at clubs, parks and open-air drug markets known to be frequented by people who use and sell illicit substances. After enrollment, participants were given project contact information and encouraged to refer other people who use substances to the study. To be eligible, participants needed to report past 30-day use of one of the focal five drugs of interest—cocaine/crack, methamphetamine, heroin, molly and bath salts. Field staff also held preliminary discussions with potential participants to ascertain English language competence and interest in the study. Following that, a screener instrument was used to establish adult age (18+) and recent incidence of illicit substance use and informed consent was collected. Staff were authorized to discontinue contact with potential participants who were felt to lack the cognitive or linguistic capacity necessary to complete the enrollment.

Quotas were established to assure adequate representation of each category of use for analytic purposes. Our data collection period coincided with changes in legal status, chemical composition and perceived risks associated with cathinone that stigmatized consumption and apparently reduced use prevalence [49]. After 4 months of recruitment, eligibility requirements for people who use cathinones were extended to the past year to assure adequate representation in the full sample.

#### Procedures

Participants were screened to establish current substance use patterns and, when eligible, were consented. Surveys were administered by experienced field staff who read each question and entered data into Android tablet computers with OpenDataKit Collect (ODK Collect) manually. Surveys were designed and scripted using Columbia University Department of Geography’s FormHub protocols and hosted online. Data were downloaded regularly and cleaned before being entered into R for the present analysis. Consent language, instruments, and all other procedures related to this study were approved by the Institutional Review board at the host agency, National Development and Research Institutes, Inc.

#### Measures

Our survey employed standard demographic variables modeled after the U.S. Census. Drug use measures included queries about ever, past year, and past 30-day use of 16 substances, including alcohol. The present analysis focuses on 9 primary drugs of abuse included in this list: bath salts (synthetic cathinones), molly, cocaine, crack, cannabis, synthetic cannabinoids, heroin, prescription drugs, and methamphetamine. Prescription drug questions focused only on non-medical use of stimulants, opioids, and benzodiazepines. Our survey also assessed past 30-day alcohol use and binge drinking using extant instrumentation which differed from significantly from the queries used for the 9 focal drugs of interest. Binge drinking was assessed with the item: “During the past 30 days, on how many days did you have 5 or more drinks on the same occasion? By ‘occasion’ we mean at the same time or within a couple of hours of each other.” The lack of parallel questionnaire language with the illicit substance questions made inclusion alongside illicit substances in the cluster analysis analytically untenable. Ultimately, we decided that a more descriptive inclusion of alcohol in relation to each individual drug of interest (rather than clusters) was the best approach.

### Approach to analysis

We approached the present analysis as an investigation of patterns of polydrug use among study participants and selected a variant of cluster analysis given the technique’s versatility in handling many forms of data and the relative conceptual simplicity of hierarchical cluster analysis (HCA), in particular, as an iterative, distance-based method [53]. HCA has been increasingly used in medical research [54–58] and studies of drug use [59–62] and is appropriate for dichotomous survey data with relatively small sample sizes (N<1000), possesses a strong bottom-up quality ideal for exploratory assessment of similarities between cases and lends itself to easily understood data visualization [63]. Specifically, an exploratory cluster analysis of use of the nine drugs was conducted by the project biostatistician, Dr. Haddock, using the “ClustOfVar” [64, 65] and “Dendextend” [66] R packages (v.3.5.1). Dendrogram plots were created where drugs with similar colored paths comprise a cluster. Our decision for the number of clusters was guided by distances in the dendrogram plots, squared loadings given different numbers of clusters, along with examining stability plots. More detail on the selection of clusters is given in S1 File.

**Fig 1:**
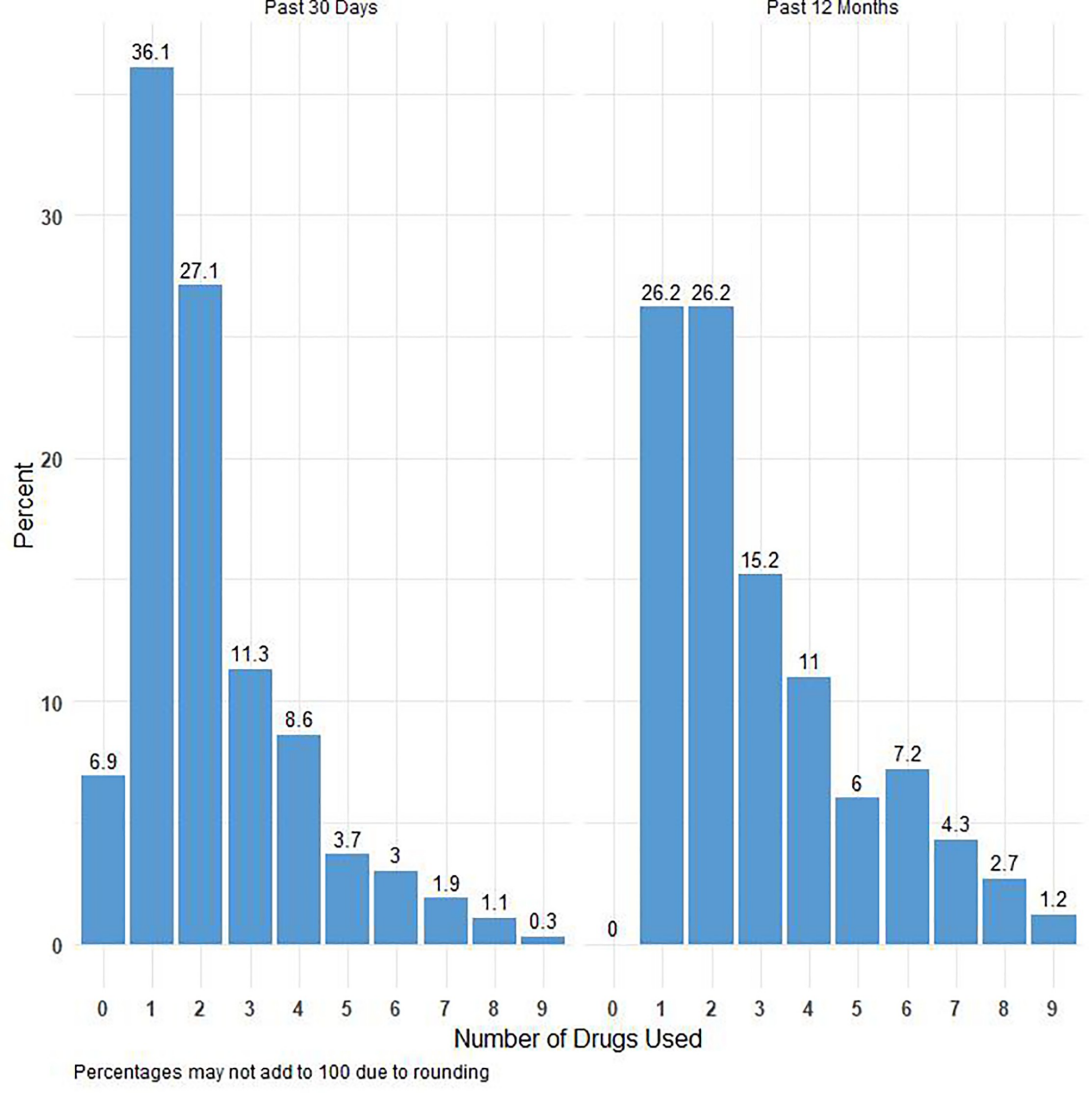
Poly drug use past 30 days and past 12 months.

Recent (30-day) and longer term (12-month) drug use prevalence, stratified by gender, minority status (white vs. other), Latino/Hispanic ethnicity, and location were derived. Only 18 individuals identified as transgender and thus were censored for stratification by gender. Ethnic minority status was determined by categorizing participants into those who identified exclusively as “white” and those who identified with one or more minority groups. We calculated chi-square statistics and p-values to examine differences among the categories. We computed the total number of the nine drugs each participant reported using over the past 30 days or 1 year. (See Fig 1) Past year bath salts users account for the entirety of the subsample reporting zero past 30-day use due to the expanded timeframe for last use of bath salts (see Sampling above).

## Results

### Participants

Participants in all three locations were predominantly low-income, with 588 of 676 (82.5%) reporting income of less than $35,000 in the past year. Table 1 presents additional background characteristics for participants by state, combining Galveston and Houston subsamples, as both cities are part of the greater Houston metropolitan area recognized by the U.S. census and participants’ referral networks often crossed between Houston and Galveston. One participant did not provide a gender identification and is not included in the Gender row. Gender balances were comparable across states (X^2^ = 1.29, p = .52). New York had a greater proportion of Latino/Hispanic participants (X^2^ = 12.3, p < .01).

**Table 1:** Participant demographics.

Demographic	All Participants (N = 676)	Interview Location		
		New York (n = 163)	Louisiana (n = 174)	Texas (n = 339)
Age Mean (SD)***	39.0 (11.7)	38.5 (12.8)	35.7 (10.1)	40.9 (11.6)
**Gender n (%)**^**†**^				
Male	372 (55.0)	92 (56.4)	97 (55.7)	183 (54.0)
Female	285 (42.2)	62 (38.0)	70 (40.2)	153 (45.1)
Transgender	18 (2.7)	8 (4.9)	7 (< 4.1)	3 (< 1.0)
Race n (%)***				
White	259 (38.3)	40 (24.5)	73 (42.0)	146 (43.1)
Black	253 (37.4)	70 (42.9)	52 (29.9)	131 (38.6)
Asian	4 (<1.0)	---	1 (< 1.0)	3 (< 1.0)
Native American	7 (1.0)	2 (1.2)	5 (2.9)	---
Multi Race	85 (12.6)	18 (11.0)	34 (19.5)	33 (9.7)
Other	68 (10.1)	33 (20.2)	9 (5.2)	26 (7.7)
Latino/Hispanic—Percent Yes*	16.9	25.2	8.6	17.1

^†^ One participant chose to not respond to the item about gender.

Note: Percentages may not add to 100 due to rounding or missing data.

Statistical significance of differences by location indicated by

* = < 0.05

** = < 0.01

*** < 0.001.

For Race percent White was compared.

### Drug use prevalence by demographics and location

Table 2 provides 30-day use prevalence rates while Table 3 contains 12-month rates for the nine drugs assessed. For both recent and past year use stratified by gender, females were more likely to use molly and prescription drugs while males were more likely to use methamphetamine. Relatively large differences were found between white participants and those from racial minority groups for most of the drug types at 30-days and 12-months. For molly, crack, and synthetic cannabis use, participants from minority groups had higher prevalence of past 30-day use while white participants had higher prevalence of prescription drug and methamphetamine use. For 12-month use, participants from minority groups had higher rates of cannabis use compared to white participants. Latino/Hispanic ethnicity was only related to the use of crack; specifically, Latino/Hispanic participants had lower prevalence at both 30-days and 12-months.

**Table 2:** Past 30-day drug use prevalence stratified by gender, minority ethnic status, and location.

% Endorsing Drug Types										
Demographic	Sample Sizes	Bath Salts	Synthetic Cannabis	Molly	Cocaine	Crack	Cannabis	Heroin	Prescription Drugs	Methamphetamine
**All**	N = 676	11.5	21.9	33.2	21.0	29.2	44.0	12.2	25.7	20.8
**Gender**^**†**^										
Male	n = 372	12.7	24.8	27.2	21.0	30.2	41.6	12.1	20.5	23.7
Female	n = 286	10.1	19.0	40.0	19.0	27.8	46.0	11.9	32.0	16.5
X^2^		0.80	2.94	11.15	0.32	0.36	1.07	<0.01	10.53	4.72
(p-value)		(0.37)	(0.09)	(<0.01)	(0.58)	(0.55)	(0.30)	(0.99)	(<0.01)	(0.03)
**Minority Status**										
White	n = 270	11.9	11.5	26.8	21.9	22.7	39.4	12.6	30.9	32.0
Minority	n = 406	11.4	28.8	37.4	20.3	33.6	47.0	11.9	22.2	13.3
Χ^2^		<0.01	27.27	7.84	0.19	8.77	3.51	0.04	5.87	32.99
(p-value)		(0.93)	(<0.01)	(<0.01)	(0.70)	(<0.01)	(0.06)	(0.85)	(0.02)	(<0.01)
**Latino/Hispanic Ethnicity**										
Latino/Hispanic	n = 114	11.5	26.3	36.8	20.4	19.5	47.8	7.1	29.2	17.0
Not L/H	n = 562	11.6	21.0	32.4	21.0	31.2	43.2	13.2	25.0	21.6
Χ^2^		<0.01	1.25	0.64	<0.01	5.70	0.62	2.74	0.68	1.02
(p-value)		(0.99)	(0.26)	(0.42)	(0.97)	(0.02)	(0.43)	(0.10)	(0.41)	(0.31)
**Location**										
New York	n = 162	4.3	40.1	35.8	30.4	31.1	65.2	13.0	24.9	13.7
Louisiana	n = 174	15.6	29.9	37.9	50.0	40.2	77.5	34.5	42.5	30.0
Texas	n = 339	13.1	9.2	29.3	1.5	22.8	16.6	<1.0	17.2	19.5
Χ^2^		11.68	69.82	4.59	174.78	17.22	211.33	125.62	38.91	14.03
(p-value)		(<0.01)	(<0.01)	(0.10)	(<0.01)	(<0.01)	(<0.01)	(<0.01)	(<0.01)	(<0.01)

^†^Given their small numbers, transgender participants were not included in the stratification by gender.

P-values are rounded to the second decimal.

Note that N’s reflect total sample size for measured demographic/location data and not participants answering each survey item.

**Table 3:** Past 12-month drug use prevalence stratified by gender, minority ethnic status, and location.

% Endorsing Drug Types										
Demographic	Sample Sizes	Bath Salts	Synthetic Cannabis	Molly	Cocaine	Crack	Cannabis	Heroin	Prescription Drugs	Methamphetamine
**All**	N = 676	21.4	30.0	49.0	33.1	36.5	50.8	16.5	35.9	29.5
**Gender**^**†**^										
Male	n = 372	20.5	32.9	42.1	33.9	38.3	48.9	17.3	30.0	32.0
Female	n = 286	22.4	26.2	55.9	31.2	33.3	51.9	14.7	42.5	25.3
X^2^		0.22	3.10	11.94	0.13	1.50	0.47	0.58	11.03	3.04
(p-value)		(0.64)	(0.08)	(<0.01)	(0.72)	(0.22)	(0.49)	(0.45)	(<0.01)	(0.08)
**Minority Status**										
White	n = 270	24.2	16.7	43.1	33.8	30.5	46.5	17.1	45.0	43.1
Minority	n = 406	19.5	38.9	53.0	32.6	40.5	53.7	16.1	30.0	20.5
Χ^2^		1.82	36.83	5.57	0.06	6.56	3.11	0.07	15.38	38.70
(p-value)		(0.18)	(<0.01)	(0.02)	(0.80)	(0.01)	(0.08)	(0.80)	(<0.01)	(<0.01)
**Latino/Hispanic Ethnicity**										
Latino/Hispanic	n = 114	21.2	38.4	54.0	34.5	28.5	53.1	10.6	42.5	26.5
Not L/H	n = 562	21.4	28.5	48.0	32.8	37.7	50.4	17.7	34.6	30.1
Χ^2^		<0.01	3.85	0.96	0.06	3.39	0.18	2.89	2.22	0.42
(p-value)		(0.99)	(0.05)	(0.33)	(0.81)	(0.07)	(0.67)	(0.09)	(0.14)	(0.52)
**Location**										
New York	n = 162	8.6	51.2	67.3	56.5	41.0	77.0	20.5	41.0	27.3
Louisiana	n = 174	28.7	43.1	32.2	71.8	54.6	84.4	44.3	56.3	46.6
Texas	n = 339	23.7	13.3	64.0	1.8	25.1	21.0	<1.0	22.8	22.0
Χ^2^		22.3	93.6	74.9	308.1	44.8	242.4	163.6	58.7	34.1
(p-value)		(<0.01)	(<0.01)	(<0.01)	(<0.01)	(<0.01)	(<0.01)	(<0.01)	(<0.01)	(<0.01)

^†^Given their small numbers, transgender participants were not included in the stratification by gender.

P-values are rounded to the second decimal.

Note that N’s reflect total sample size for demographic/location and not participants answering each survey item.

Use of the nine drug types differed markedly by location. Participants from our Texas sites tended to have lower prevalence of use except for bath salts and molly. Prevalence of bath salts use was particularly low among participants from the New York area while very few participants from Texas reported using cocaine or heroin either in the past 30-days or 12-months.

### Prevalence rates of poly drug use

Fig 1 presents the prevalence of poly drug use for both 30-day and 12-month use. The largest proportion of participants used either one (36.1%) or two (27.1%) drugs in the past 30 days. The most common drugs used by those who reported only using one drug in the past 30 days was crack (21.5%) followed closely by molly (21.1%). For those reporting using two drugs in the past 30 days, the most common were cannabis (52.2%) and molly (36.8%) For use in the past 12 months, the highest proportion of participants used either one (26.2%) or two drugs (26.2%). The most common drugs used by those who used one drug in the past 12 months were molly (26.7%) and crack (22.7%). For those reporting two drugs in the past 12 months, the most common were cannabis (38.1%) and molly (36.4%).

### Cluster analysis of drug use

**Fig 2:**
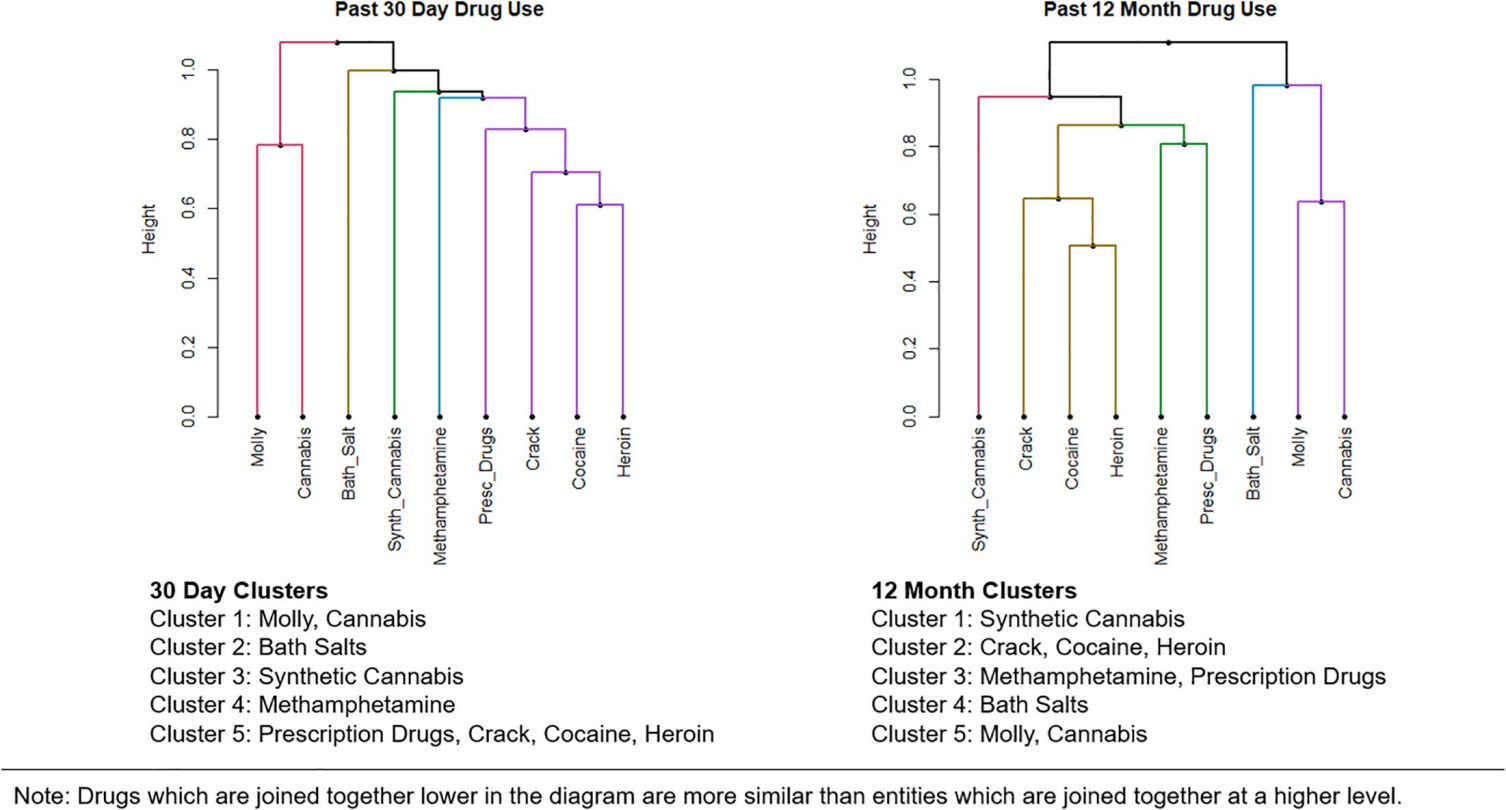
Cluster analysis of past 30-day and past 12-month drug use.

Fig 2 contains dendrogram plots of use of the nine drugs across 30-days and 12-months. Horizontal branches, or “clades” on dendrograms represent clusters of data points that are more similar to each other than to those in other clusters. Clades can have one or more leaves which represent the items in a cluster, or in this paper the drugs under consideration. The greater the height at which they are joined, the greater the difference between entities joined on a branch. Thus, entities which are joined together lower in the diagram are more similar than entities which are joined together at a higher level. Dissimilarity between data points in different clusters was calculated using Jaccard distance given the binary nature of the primary variables of interest.

A 5-cluster solution was chosen for both time periods after consideration of 4-, 5-, and 6-cluster solutions (see S1 File). The 5-cluster model provided both an appealing cut on the dendrogram and relatively strong cluster loadings (all > 0.5). Increasing to 6 factors neither appreciably decreased the cut point on the dendrogram nor improved cluster loadings. Stability and aggregation plots suggested 6 clusters with 5 clusters as a next possible choice. The choice between 5 and 6 clusters was a choice of representing methamphetamine and prescription drugs in one or individual clusters. However, given that these two drugs had relatively strong loadings (>0.60) when joined in a 5-cluster solution, a visual analysis of the dendrogram (i.e., the two drugs were located on a common branch), and the fact that it is reasonable that methamphetamine and prescriptions drugs co-occur, a 5-cluster model was selected.

Two clusters of drugs in particular exhibited strong consistency across the past 30-day and past 12-month plots. Molly and cannabis use were related for both time periods as were crack, cocaine, and heroin. Prescription drugs was associated with crack, cocaine, and heroin use for past 30-day use but only with methamphetamine for past 12-month use. Bath salts and synthetic cannabis were not significantly associated with use of the other drugs at either time period. When a one-cluster solution was explored with the data, bath salts was the least related to the other 8 drugs for both time periods.

### Use of alcohol by other drug use

**Fig 3:**
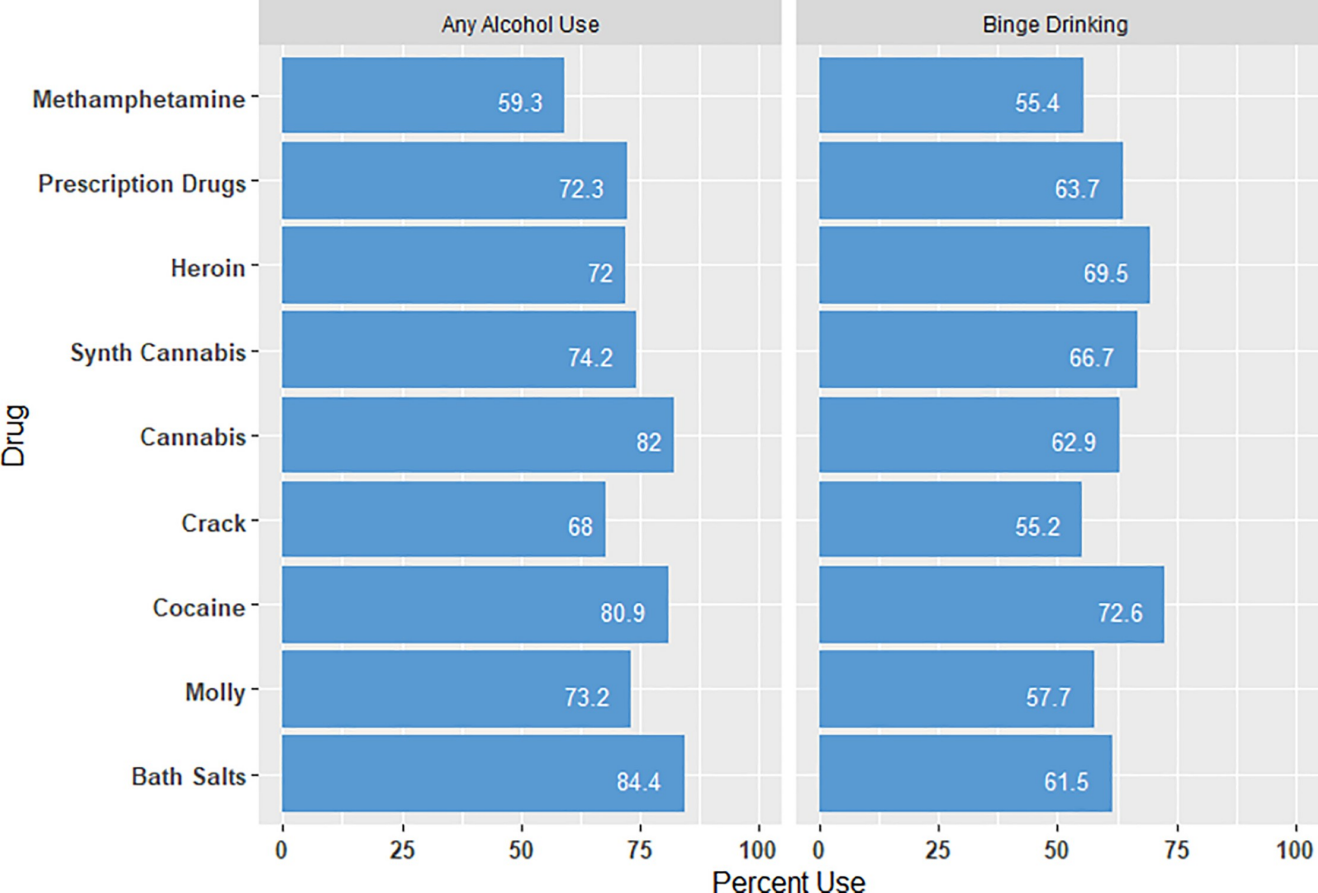
30-Day drug use and alcohol consumption.

Past 30-day alcohol consumption (see Fig 3) was highly prevalent among the participants, with use increasing with a larger number of drugs used in the same time frame. Among those using 0 to 1 drug in the past 30 days, 56.6% reported any alcohol use, while among those who reported using 2 to 4 drugs 73.1% drank alcohol and for those using 5 or more drugs 82.1% drank. Prevalence of alcohol use and binge drinking (≥5 drinks in one occasion) was high (i.e., more than half of all users) for all drug types. The highest prevalence of alcohol use was among cannabis and bath salts users, with methamphetamine users reporting the lowest consumption of alcohol. For binge drinking, cocaine users had the highest prevalence followed by heroin and prescription drug users.

## Discussion

We undertook this analysis to examine patterns of polysubstance use and the particular place of novel synthetic substances (here, those chemically related to the active ingredients in *cannabis* and cathinones). We found substantial differences in substance use patterns by demographics and location. Perhaps the most salient finding in this study relates to the relative lack of clustering among novel synthetic substances (bath salts and K2/Spice categories of drug analogues, specifically) and any of 7 other scheduled drugs. Our finding that conventional cannabis and its synthetic analogues are not commonly used interchangeably by the same individuals suggests either that the substance effects are sufficiently different to appeal to different populations of people who use substances for different reasons, or that synthetic cannabinoids are used out of a perceived necessity, not preference. Some published literature suggests that synthetic cannabinoids are used often as a substitution for cannabis by those seeking to avoid positive urinalysis in the workplace or as a condition of their parole or probation [37]. Other work has established the commonplace use of these substances among low-income and homeless populations, in particular [67]. Both of these trends suggest that the potentially deleterious side-effects [20, 22–24] of an understudied class of more than 100 chemical analogues are much more likely to impact already vulnerable populations. Educational and harm reduction efforts to engage these populations productively urge individuals to take extremely small “test doses”—akin to the test shots utilized by people who use heroin to gauge potency and OD risk [68]—before smoking their normal quantity of K2/Spice.

In the case of bath salts, our finding that cathinones were not clearly clustered with any other drug category was unexpected. Literature suggests that some of the more popular cathinone analogues are held to be experientially close to amphetamine-class substances—some resembling methamphetamine and others closer to 3-4-Methylenedioxymethamphetamine (MDMA, or molly/Ecstasy) in their effects [49, 69]. As such we expected a clear clustering of use with molly and methamphetamine that would be evident in the dendrograms above and suggestive of a substitution process whereby people who use one substance turn to another with comparable effects when impacted by scarcity, diminished quality, heightened tolerance, or economic pressures to consider cheaper alternatives. In the absence of such findings, we propose a pair of speculative interpretations that may bear further research. First, the seeming lack of coherent polysubstance use patterns surrounding bath salts may be a product of the pharmacological diversity within the synthetic cathinones. Regional market-level differences (combined with variant subcultural use patterns) could produce adequate pharmacological diversity to explain the wide variety of other substances consumed by people who use bath salts—a case of many different cathinones for many different types of people who use substances. Second, the lack of clustering between synthetic cathinones and other substances may be driven by the relative novelty of the drug category. As some drug theorists have argued, drug trends have a kind of natural history, beginning with widespread experimentation by a broad cross-section of the substance using population. During this experimental period, it may be hard to predict the subcultural niche into which a novel substance will fit once some cohorts of experimenters become more regular users [70, 71]. Given that synthetic cathinones (and synthetic cannabinoids) have only recently moved from the grey market to fully scheduled illegal drugs, the absence of clear polysubstance clustering grounded in drug subculture formation is perhaps to be expected. Ongoing monitoring of the use of these potentially deleterious substances and the changing analogues being introduced to the illicit marketplace will be critical to avoiding undue public health impact.

Despite the lack of clear indications that synthetic use follows a substitution or replacement logic within more established patterns of substance (or polysubstance use), our analyses did identify a number of clear patterns of polysubstance use. Across sites, we found less polysubstance use than might have been anticipated from recent literature on emerging patterns of substance use in the U.S. [1]. On past 30-day use measures, the two largest groups of participants used either one or two drugs. For those only using one drug, crack and molly were the most common. For use in the past 12 months, the highest proportion of study participants reported using either one or two drugs. Those who consumed only one drug were more likely to use molly or crack; those who consumed two drugs were more likely to use cannabis and molly, a finding likely grounded in the strong association between the two in “club culture” [72–74] and the potentially protective effects of THC on MDMA-related hyperthermia [75]. Our cluster analysis of drug use across both 30 days and 12 months showed relationships between two sets of drugs: molly/cannabis and crack/cocaine/heroin. Substance use across 30 days points to relationships between prescription drugs and crack, cocaine and heroin use. Additionally, we found relatively high levels of past month drinking across almost all forms of illicit substance use. Extremely high proportions of cocaine and heroin users who reported any drinking in the past 30-days also reported binge drinking. These findings generally reinforce the need for ongoing efforts to better address the highly significant role of overlapping alcohol and opioid use disorders in opioid overdose mortality [76] as well as the role of concurrent cocaine/crack use upon dependence and toxicity [12, 14, 39].

Participants identifying as members of one or more racial minority groups—predominantly Black/African-American in our sample—had higher past 30-day prevalence rates of molly, crack, and synthetic cannabis use, while white participants had higher prescription drug and methamphetamine use. Minority participants also reported significantly higher past 12-month use of marijuana/cannabis. Latino/Hispanic ethnicity was not significantly related to substance use patterns except for crack use. Latino/Hispanic participants reported significantly less past 30 and past 12-month crack use than their non-Latino/Hispanic counterparts. Regionally, we found marked differences between participants in Texas, Louisiana, and New York. Participants from our Texas sites had lower prevalence of use across most drug categories, with the exception of bath salts and molly. Rates of bath salts use were particularly low among participants from the New York area while very few participants from the greater Houston area reported using cocaine or heroin either in the past 30-days or 12-months. These findings broadly demonstrate the ongoing importance of local monitoring efforts that are sensitive to regional and cultural differences in use and the potential for differential involvement with particular forms of substance use and related harms by race and other background factors.

## Limitations

Detailed understanding of polysubstance use patterns and attendant health risks requires a focus on concurrency that lay beyond the scope of this research. While the analysis is strengthened by both past 30-day and past year measurement of drug use as proxies for concurrent polysubstance use, future research into the place of synthetics within typologies of polysubstance use would benefit from more fine-grained measurement of overlapping use, potentially via timeline follow-back or ecological momentary assessment data collection methods. Steering strategies employed to assure adequate representation of bath salts users in the sample represent an inconsistency that may serve to overstate the relationship between bath salts and alcohol relative to the other drug + alcohol combinations. While the decision to enroll past year bath salts users (a less stringent criterion than past 30 days) had the potential to limit the power necessary to establish bath salt-related clustering, both past year and past 30-day findings were aligned, indicating a lack of discernible clustering with any other specific substances. Non-probability sampling allowed for targeted recruitment of synthetic drug users, but came at the expense of our ability to infer to population-level estimates of synthetic drug use or polysubstance use by state or region. As regional patterns of substance use may involve different patterns of polysubstance use, the findings here are largely preliminary. Larger, regionally-sensitive samples should be employed to understand emerging polysubstance use patterns and related harms in localized areas and should examine how membership in distinct polysubstance clusters are correlated with a range of health outcomes. Outcomes involving criminal justice involvement and hospitalization in our study were linked to particular drug queries and thus ill-suited to analysis in terms of the cluster analysis presented. Finally, as with all substance-related research involving self-report data, participants may have underestimated their substance use. Carefully trained field staff experienced in non-stigmatizing rapport-building with people who use drugs in this study should have served to mitigate underestimation due to social desirability, however.

## Conclusions

Findings suggest considerable variation in substance and polysubstance use patterns across states in the U.S. Polysubstance patterns among people who use scheduled drugs were not as common as anticipated and polysubstance use tended to cluster around traditional, culturally established combinations like MDMA/cannabis and cocaine/heroin. In our analysis novel synthetic cannabinoids and cathinones (that is, “K2/spice” and “bath salts” type substances) showed no strong clustering with other substances, suggesting that dominant subcultural polysubstance patterns have yet to emerge and/or that pharmacological variation within these emergent categories has resulted in widely divergent interests in them among different types of people who use illicit substances. The near universality of drinking and high rates of binge drinking among users of other substances are evidence of the importance of addressing concurrent alcohol and other substance use with treatments and interventions sensitive to the clinical challenges of problematic polysubstance use. Findings generally demonstrate the importance of monitoring the impact of socioeconomic factors on novel psychoactive substance use and the potential for emergent, relatively low-cost synthetic analogue drug markets to represent the greatest harm to more historically disadvantaged populations of people who use drugs.

## Supporting information

S1 File Supporting materials. (Figure A) Dendrograms representing 4, 5, and 6 clusters. (Table A) Squared loadings for 4 clusters. (Table B) Squared loadings for 5 clusters. (Table C) Squared loadings for 6 clusters.
(DOCX)

S1 Dataset Polysubstance clusters dataset. (XLSX)

S1 Codebook Polysubstance clusters codebook. (DOCX)
