# Supplementary material for: Polysubstance use patterns and novel synthetics: A cluster analysis from three U.S. cities
Source: PLoS One. 2019 Dec 3;14(12):e0225273. doi: 10.1371/journal.pone.0225273 (PMC6890248; doi:10.1371/journal.pone.0225273)
Supplement: S1 File — (Figure A) Dendrograms representing 4, 5, and 6 clusters. (Table A) Squared loadings for 4 clusters. (Table B) Squared loadings for 5 clusters. (Table C) Squared loadings for 6 clusters. (DOCX) [file pone.0225273.s001.docx]

Supporting information

Rationale for selection of 5-cluster solution

Similar to determining the number of factors to extract from an exploratory factor analysis, selecting the number of clusters is somewhat ambiguous, depending on both the judgement of the investigators and distribution of variables in the analysis. Because we are not testing whether a specific model (or competing models) fit the data (as in confirmatory factor analysis), we used a combination of visual and analytical tools to determine the number of clusters in the data. Our primary three methods for selecting the number of clusters was examining the dendrogram, comparing different “cuts” on the dendrogram to the factor loadings given a particular selection of clusters, and having our topical experts (LE, EB) judge the interpretability of the different choices. Dendrograms for Past 12-month drug use are given in Figure 4. The three dendrograms reflect three different cut points representing 4, 5, and 6 clusters (cut points represented by horizontal bars). The lower on the y-axis the analyst draws a horizontal line (i.e., the distances) the larger number of clusters will be selected. Drugs which cluster closer together lower in the y-axis are closer in distance than those which cluster higher on the graph. We have juxtaposed the graphs with tables of the squared cluster loadings given the three different choices.

**Fig A. Dendrograms Representing 4, 5, and 6 Clusters**

**
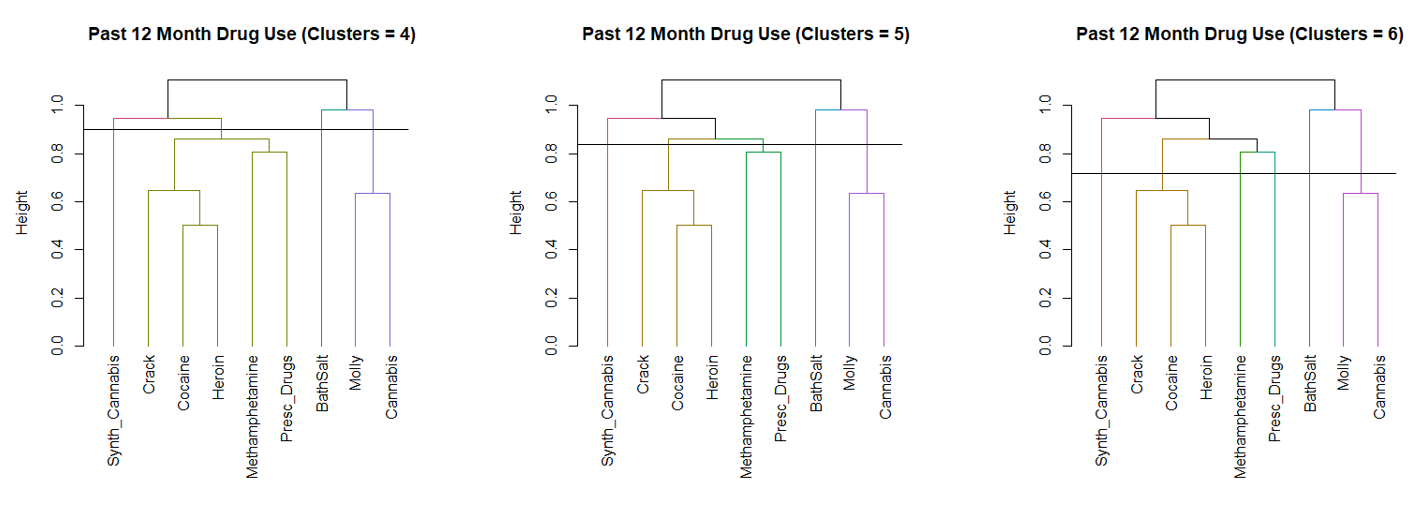
**

Carefully examining the dendrograms for different potential solutions, we arrived at the best three cluster loadings for potential solutions (see Tables A, B, C). In the factor loadings for 4 clusters both methamphetamine and prescription drugs were clearly not as related to the other drugs in cluster 3. The 5-cluster model provided both an appealing cut on the dendrogram and relatively strong cluster loadings (all > 0.5). Increasing to 6 factors neither appreciably decreased the cut point on the dendrogram nor improved cluster loadings. In fact, a strong case could be made from the plot and squared loadings that methamphetamine and prescriptions drugs cluster together. The stability and aggregation plots suggested 6 clusters with 5 clusters as a next possible choice. The choice between 5 and 6 clusters was a choice of representing methamphetamine and prescription drugs in one or individual clusters. However, given the cluster loadings, a visual analysis of the dendrogram (i.e., the two drugs were located on a common branch), and the fact that it is reasonable that methamphetamine and the broad category of prescription drug misuse co-occur, a 5-cluster model was selected.

**Table A: Squared Loadings for 4 Clusters**

| **Cluster** | **Loading** |
| --- | --- |
| *Cluster 1* |  |
| Synthetic Cannabis | 1 |
| *Cluster 2* |  |
| Molly | 0.68 |
| Cannabis | 0.68 |
| *Cluster 3* |  |
| Cocaine | 0.62 |
| Heroin | 0.61 |
| Crack | 0.40 |
| Methamphetamine | 0.30 |
| Prescription Drugs | 0.25 |
| *Cluster 4* |  |
| Bath Salts | 1 |

**Table B: Squared Loadings for 5 Clusters**

| **Cluster** | **Loading** |
| --- | --- |
| *Cluster 1* |  |
| Synthetic Cannabis | 1 |
| *Cluster 2* |  |
| Molly | 0.68 |
| Cannabis | 0.68 |
| *Cluster 3* |  |
| Cocaine | 0.63 |
| Heroin | 0.67 |
| Crack | 0.55 |
| *Cluster 4* |  |
| Methamphetamine | 0.60 |
| Prescription Drugs | 0.60 |
| *Cluster 5* |  |
| Bath Salts | 1 |

**Table C: Squared Loadings for 6 Clusters**

| **Cluster** | **Loading** |
| --- | --- |
| *Cluster 1* |  |
| Synthetic Cannabis | 1 |
| *Cluster 2* |  |
| Molly | 0.68 |
| Cannabis | 0.68 |
| *Cluster 3* |  |
| Cocaine | 0.63 |
| Heroin | 0.67 |
| Crack | 0.55 |
| *Cluster 4* |  |
| Methamphetamine | 1 |
| Cluster 5 |  |
| Prescription Drugs | 1 |
| *Cluster 6* |  |
| Bath Salts | 1 |
