# Supplementary material for: Polysubstance use patterns and novel synthetics: A cluster analysis from three U.S. cities
Source: PLoS One. 2019 Dec 3;14(12):e0225273. doi: 10.1371/journal.pone.0225273 (PMC6890248; doi:10.1371/journal.pone.0225273)
Supplement: S1 Codebook — (DOCX) [file pone.0225273.s003.docx]

**Computer Assisted Personal Interview**

**Data Dictionary**

| **Variable**  **Number** | **Variable Name** | **Item** | **Response Set** | **Skip Pattern** |
| --- | --- | --- | --- | --- |
| 001 | INTVIEWID | Interviewer Last Name | Alpha Numeric Text Field |  |
| 002 | INTVIEWDATE | Date of interview | mm/dd/yyyy |  |
| 003 | INTVIEWCITY | City of Interview | 1=New Orleans City  2=New Orleans Area  3=Elsewhere in LA  4=City of Houston TX  5=Harris Suburbs and County  6=Galveston TX  7=Elsewhere in SE TX  8=New York City  9=Other |  |
| 003a | OTHERCITY | Other location of interview | Alpha Numeric Text Field |  |
| 004 | RESPID | Respondent’s ID number | C + 3 Digit Identifier (e.g., C251) |  |
| 005 | GENDER | First I would like to ask you a couple of questions about yourself. Are you Male, Female, or Transgender? | 1=Male  2=Female  3=Transgender  97=Didn’t Answer  98=Refused to Answer |  |
| 006 | BYEAR | What year were you born? | yyyy |  |
| 007 | BMONTH | What month were you born? (numeric from 01 to 12) | mm |  |
| 008 | HISPANIC | Are you of Hispanic, Latino, or Spanish origin? (the US Census, Hispanic origins are not races) | 0=No, not of Hispanic, Latino, or Spanish Origin  1=Yes, Mexican, Mexican American, Chicano  2=Yes, Puerto Rican  3=Yes, Cuban  4=Yes, another Hispanic, Latino, or Spanish origin)  97=Don’t know  98=Refused to answer | If 010 NE 4 (another Hispanic, Latino, or Spanish origin), skip to 010 |
| 009 | OTHHISPANIC | If Other Hispanic, Latino, or Spanish origin selected, please ask them to provide origin (e.g., Argentinian, Columbian, Dominican, Nicaraguan, Salvadoran, Spaniard, and so on) and enter other Hispanic, Latino, or Spanish origin. > | Alpha Numeric Text Field |  |
| For 010 to 017 ask: How would you describe your racial background? Read each race option and have them select all that they identify with. | | | | |
| 010 | RACEWHITE | Are you White/Caucasian? | 0=No  1=Yes  97=Don’t Know  98=Refused to Answer |  |
| 011 | RACEBLACK | Are you Black/African American? | 0=No  1=Yes  97=Don’t Know  98=Refused to Answer |  |
| 012 | RACENATIVEAM | Are you American Indian or Alaskan Native? | 0=No  1=Yes  97=Don’t Know  98=Refused to Answer |  |
| 013 | RACEASIAN | Are you of Asian origin? | 0=No, not of Asian origin  1=Yes, Asian Indian  2=Yes, Chinese  3=Yes, Filipino  4=Yes, Japanese  5=Yes, Korean  6=Yes, Vietnamese  7=Yes, another Asian origin  97=Don’t Know  98=Refused to Answer | If NE 7 (another Asian origin), skip to 015 |
| 014 | RACEOTHERASIAN | Other Asian: If selected, please ask them to provide what race (e.g., Hmong, Laotian, Thai, Pakistani, Cambodian, and so on) and enter other Asian race. | Alpha Numeric Text Field |  |
| 015 | RACENATIVEHAWIAN | Are you of Native Hawaiian or other Pacific Islander origin? | 0=No  1=Yes, Native Hawaiian  2=Yes, Guamanian or Chamorro  3=Yes, Samoan  4=Yes, other Pacific Islander  97=Don’t Know  98=Refused to Answer | If NE4 (other Pacific Islander), skip to 017 |
| 016 | RACEOTHERPI | Other Pacific Islander: If selected, please ask them to provide what race (e.g., Fijian, Tongan, and so on) and enter other Pacific Islander race | Alpha Numeric Text Field |  |
| 017 | RACEOTHER | Are you another race? | Alpha Numeric Text Field |  |
| 018 | RACEWHICH | Other race: If selected, enter other race | Alpha Numeric Text Field |  |
| 019 | EDUCATE | What is the highest educational degree you have? | 1=Grade School or No Education  2=Middle School/Junior High  3=Didn’t Finish High School  4=High School or GED  5=Some College  6=Two Year Associates Degree  7=Trade or Vocational School  8=Four Year College Degree  9=Graduate Degree  97=Don’t Know  98=Refused to Answer |  |
| 020 | RELATIONSHIP | Are you now married, widowed, divorced or separated? | 1=Currently Married  2=Widowed  3=Divorced or Separated  4=Never married  97=Don’t Know  98=Refused to Answer |  |
| 021 | WHERELIVE | During the past 30 days, where have you lived most of the time? | 1=House  2=Apartment  3=Hotel or motel  4=Condominium or Co-Op  5=Trailer or Mobile Home  6=Home of Relative  7=Home of Friend  8=Shelter  9=Medical Facility  97=Don’t Know  98=Refused to Answer |  |
| 022 | MYCHILD | Do you have children? | 0=No  1=Yes  97=Don’t Know  98=Refused to Answer  99=Not Applicable |  |
| 023 | OWN | Do you own your current residence? | 0=No  1=Yes  97=Don’t Know  98=Refused to Answer  99=Not Applicable |  |
| 024 | ZIPCODE | What is your current ZIP Code? | F5.0 |  |
| 025 | INCOMESRC | What is your primary source of income? | 1=Employment  2=Public Benefits  3=Family/friends  4=Off-books work or legal hustles  5=Illegal hustles  6=Other  97=Don’t Know  98=Refused to Answer  99=Not Applicable |  |
| 026 | INCOME | What is your approximate income from all sources? | 1=<$25,000  2=$25,000 to $34,999  3=$35,000 to $49,999  4=$50,0000 to $74,999  5=>$75,000  97=Don’t Know  98=Refused to Answer  99=Not Applicable |  |
| 027 | SLFRATHEALTH | This question is about your overall health. Would you say your health in general is excellent, very good, good, fair, or poor? | 1=Excellent  2=Very Good  3=Good  4=Fair  5=Poor  97=Don’t Know  98=Refused to Answer |  |
| 028 | BATHSALT | Have you ever, even once, used bath salts? | 0=No  1=Yes  97 = Don’t know  98 = Refused to answer | If NE 1(Yes) skip to 95 |
| 029 | BSWHEN | How long has it been since you last used bath salts? | 1=Within past 30 days  2=More than 30 days ago but within the past 12 months  3=More than 12 months ago  97=Don’t Know  98=Refused to Answer | If 3 (More than 12 months ago), skip to 95 |
| 030 | PASTYRBSDAYS | On average, how many days did you use bath salts each month in the past 12 months? | F2.0  97=Don’t Know  98=Refused to Answer |  |
| 031 | PAST30BSDAYS | Think specifically about the past 30 days, from [DATE] up to and including today. On how many of those days did you use bath salts? | F2.0  97=Don’t Know  98=Refused to Answer |  |
| 032 | BSLEARN | How did you learn about bath salts? | 1 = Relative  2 = Partner or lover  3 = Work associate  4 = Friend/Acquaintance 5 = Dealer  6 = Media  7 = Other  97 = Don’t know  98 = Refused to answer | If NE 7 (Other) Skip to 034 |
| 033 | BSLEARNOTHER | What other way did you learn about bath salts? | Alpha Numeric Text Field |  |
| 034 | BSNUMFRIEND | How many of your friends would you estimate use bath salts? | 1 = None  2 = A few  3 = Some  4 = Most  5 = All  97 = Don’t know  98 = Refused to answer |  |
| 035 | BSHOWPRIMARY | What is your primary way of using bath salts? | 1=Smoke  2=Swallow  3=Snort  4=Inject  5=Other  97=Don’t Know  98=Refused to Answer | If NE 5 (Other) skip to 037 |
| 036 | BSHOWOTHER | Explain the “other” way you use bath salts | Alpha Numeric Text Field |  |
| 037 | BSHOWSECOND | What is your secondary way of using bath salts? | 1=Smoke  2=Swallow  3=Snort  4=Inject  5=Other  6=No secondary method  97=Don’t Know  98=Refused to Answer | If NE 5 (Other) skip to 039 |
| 038 | BSHOWOTHSEC | Explain the “other” secondary way you use bath salts | Alpha Numeric Text Field |  |
| 039 | BSALCOHOL | Do you use bath salts with alcohol? | 0=No  1=Yes  97=Don’t Know  98=Refused to Answer |  |
| 040 | BSOTHDRUG | Do you use bath salts with other drugs? | 0=No  1=Yes  97=Don’t Know  98=Refused to Answer | If 0(No) skip to |
| 041 | BSOTHDRUGD | What other drugs do you use with bath salts? | Alpha Numeric Text Field |  |
| 042 | BSWHERE | Where do you usually use bath salts? | 1=Home, apartment, or dorm  2=Friend’s Home  3=Parties  4=Concerts/Festivals  5=Clubs/Bars  6=Park, nature, outdoor  7=On the street, sidewalk  8=Other  97=Don’t Know  98=Refused to Answer | If NE 8(Other), skip to |
| 043 | BSWHEREOTHR | In what “other” location do you typically use bath salts? | Alpha Numeric Text Field |  |
| 044 | BSALONE | Do you usually use bath salts by yourself or with other people? | 1=Alone  2=With Other People | If 1 (Alone) skip to 046 |
| 045 | BSWITHWHO | Who do you usually use bath salts with? | Alpha Numeric Text Field |  |
| 046 | BSWHY | What is your primary reason for using bath salts? | 1=To Experiment – See What It’s Like  2=To Relax  3=To Feel Good or Get High  4=To increase sexual arousal and/or performance  5=To Have a Good Time with Friends  6=To Get Away from My Problems or Troubles  7=Because of Boredom, Nothing Else to Do  8=Because of Anger or Frustration  9=To Increase the Effect of Some Other Drug  10=To Decrease/Offset the Effects of Some Other Drug  11=Because I am “Hooked” – I Have to Have It  12=Other  97=Don’t Know  98=Refused to Answer | If NE 12 (Other) skip to |
| 047 | BSWHYOTHER | What is your “other” primary reason for using bath salts? | Alpha Numeric Text Field |  |
| For the next few questions I’m going to ask you whether you have had certain problems because of your use of bath salts. | | | | |
| 048 | BSREGRET | Has bath salts caused you to do things you later regretted? | 0=No  1=Yes  97=Don’t Know  98=Refused to Answer |  |
| 049 | BSHURTSCH | Has your use of bath salts hurt your performance in school and/or on the job? | 0=No  1=Yes  97=Don’t Know  98=Refused to Answer |  |
| 050 | BSPOLICETROB | Has your use of bath salts caused you to get in trouble with the police? | 0=No  1=Yes  97=Don’t Know  98=Refused to Answer |  |
| 051 | BSPROBOTHER | Has your use of bath salts caused you other problems? | 0=No  1=Yes  97=Don’t Know  98=Refused to Answer | If 0 (No) Skip to |
| 052 | BSPROBOTHERO | What other problems has your use of bath salts caused you? | Alpha Numeric Text Field. |  |
| 053 | BSALOT | During the past 12 months, was there a month or more when you spent a lot of your time getting or using bath salts? | 0=No  1=Yes  97=Don’t Know  98=Refused to Answer |  |
| 054 | BSGETOVER | During the past 12 months, was there a month or more when you spent a lot of your time getting over the effects of the bath salts you used? | 0=No  1=Yes  97=Don’t Know  98=Refused to Answer |  |
| 055 | BSEFFECTS | During the past 12 months, did you need to use more bath salts than you used to in order to get the effect you wanted? | 0=No  1=Yes  97=Don’t Know  98=Refused to Answer |  |
| 056 | BSCTDWN | During the past 12 months, did you want to or try to cut down or stop using bath salts? | 0=No  1=Yes  97=Don’t Know  98=Refused to Answer | If 0 (No) skip to |
| 057 | BSCTDWNALO | During the past 12 months, did you cut down or stop using bath salts at least one time? | 0=No  1=Yes  97=Don’t Know  98=Refused to Answer | If 0 (No) skip to |
| 058 | BSSYMPTOMS | Please look at these symptoms. During the past 12 months, did you have any of these symptoms after you cut back or stopped using bath salts?  *Feeling tired or exhausted  *Having bad dreams  *Having trouble sleeping or sleeping more than usual  *Feeling blue or depressed  *Feeling hungry more often  *Feeling either very slowed down or like you couldn’t sit still | 0=No  1=Yes  97=Don’t Know  98=Refused to Answer |  |
| 059 | BSWORSE | During the past 12 months, did you have any problems with your emotions, nerves, or mental health that were caused or made worse by using bath salts? | 0=No  1=Yes  97=Don’t Know  98=Refused to Answer | If 0 (No) skip to |
| 060 | BSUSEANYWAY | Did you continue to use bath salts even though you thought it was causing you to have problems with your emotions, nerves, or mental health? | 0=No  1=Yes  97=Don’t Know  98=Refused to Answer |  |
| 061 | BSHLTSPROB | During the past 12 months, did you have any physical health problems that were probably caused or made worse by your use of bath salts? | 0=No  1=Yes  97=Don’t Know  98=Refused to Answer | If 0 (No) skip to |
| 062 | BSHLTHPROBA | Did you continue to use bath salts even though you thought it was causing you to have physical health problems? | 0=No  1=Yes  97=Don’t Know  98=Refused to Answer |  |
| 063 | BSPROBFAM | During the past 12 months, did you have any problems with family or friends that were probably caused by your use of bath salts? | 0=No  1=Yes  97=Don’t Know  98=Refused to Answer | If 0(No) skip to |
| 064 | BSPROBFAMA | Did you continue to use bath salts even though you thought it caused problems with family or friends? | 0=No  1=Yes  97=Don’t Know  98=Refused to Answer |  |
| 065 | BSFIGHT | Have you ever gotten into a fight or done anything violent after using bath salts? | 0 = No  1 = Yes  97 = Don’t Know  98 = Refused to Answer |  |
| 066 | BSVICTIM | Have you ever been a victim of violence while using bath salts? | 0 = No  1 = Yes  97 = Don’t Know  98 = Refused to Answer |  |
| 067 | BSVIOLENCE | Have you ever seen anyone become violent while using bath salts? | 0 = No  1 = Yes  97 = Don’t Know  98 = Refused to Answer |  |
| 068 | BSER | Have you ever gone to an emergency room after using bath salts? | 0=No  1=Yes  97=Don’t Know  98=Refused to Answer |  |
| 069 | BSPOISONC | Have you ever called a poison control center after using bath salts? | 0=No  1=Yes  97=Don’t Know  98=Refused to Answer |  |
| 070 | BSOBTAIN | Now think about the last time you used bath salts. How did you get these bath salts? | 1 = Bought it  2 = I traded something else for it  3 = I got it for free or shared someone else’s  4 = I made it myself  97 = Don’t know  98 = Refused to answer |  |
| 070b | OBTAINOTH | What other way did you get bath salts? | Alpha Numeric Text Field |  |
| 071 | BSFORM | What form of bath salts did you acquire the last time you used bath salts? | 1 = Powder  2 = Crystals  3 = Other  97 = Don’t Know  98 = Refused to answer | If NE 4 (Other) Skip to |
| 072 | BSFORMOTH | What “other” form of bath salts did you use? | Alpha Numeric Text Field |  |
| 073 | BSUNIT | What was the unit type of bath salts that you received the last time you used bath salts? | 1 = Small bag or sack  2 = Vials/small jars or bottles  3 = Grams  4 = Ounces  5 = Other  97 = Don’t Know  98 = Refused to answer | If NE 5 (Other) Skip to |
| 074 | BSUNITOTHER | What “other” unit type did you receive? | Alpha Numeric Text Field |  |
| 075 | BSGPRICE | In general, what do bath salts cost? [Price in dollars] | F5.2 |  |
| 076 | BSGPRICEUNIT | What is the unit of the general price for bath salts? | 1 = Small bag or sack  2 = Vials/small jars or bottles  3 = Grams  4 = Ounces  5 = Other  97 = Don’t Know  98 = Refused to answer | If NE 3 (Grams) or 4 (Ounces) Skip to |
| 077 | BSOTHERUNIT | What “other” unit? | \| Alpha Numeric Text Field \| \| --- \| \|  \| \|  \| \|  \| \|  \| \|  \| \|  \| |  |
| 078 | BSBUY | Have you ever bought bath salts yourself? | 0 = No  1 = Yes  97 = Don’t know  98 = Refused to answer | If NE 1(Yes), Skip to |
| 079 | BSLTWHO | The last time you bought bath salts, who did you buy it from? | 1 = A friend/acquaintance  2 = A relative or family member  3 = Someone I had just met or didn’t know well  4 = The Internet  5 = Delivery service  6 = Store, bodega or smoke shop  7 = Dealer  8 = Other  97 = Don’t know  98 = Refused to answer | If NE 8 (Other) Skip to |
| 080 | BSLTWHOO | What “other” source did you buy bath salts from? | Alpha Numeric Text Field |  |
| 081 | BSLTBUYWHERE | The last time you bought bath salts, where were you when you bought it? | 1 = At a club, bar or festival  2 = At a school  3 = At a home, apartment, or dorm  4 = Outside in a public area (e.g., street or park)  5 = Other  97 = Don’t know  98 = Refused to answer | If NE 6 (Other) Skip to |
| 082 | BSLTBUYWHEREO | What “other” location were you in the last time you bought bath salts? | Alpha Numeric Text Field |  |
| 083 | BSLTBUYRELATION | What is your relationship with your usual bath salts source? | 1 = Relative  2 = Partner or lover  3 = Work associate  4 = Friend/Acquaintance 5 = Dealer (who is not a friend/relative/partner)  6 = No personal relationship  97 = Don’t know  98 = Refused to answer |  |
| 084 | BSLTBUYUNIT | The last time you bought bath salts, what units did you buy it in? | 1 = grams  2 = ounces  3 = bags  4 = vials/small jars or bottles  5 = other  97 = Don’t know  98 = Refused to answer | If 6 (Did not buy bath salts) skip to  92  If 97 or 99, skip to 87  Else if NE 5 (Other) skip to 86 |
| 085 | BSLTBUYUNITO | The last time you bought bath salts what “other” unit did you buy it in? | Alpha Numeric Text Field |  |
| 086 | BSLTBUYAMT | The last time you bought bath salts, how many gms/ozs/bags/vials/other did you buy? [Use the unit respondent provided in previous question] | F5.2 |  |
| 087 | BSLTMONEY | The last time you bought bath salts, how much money did you spend? (whole dollars) | F4.0 |  |
| 088 | BSLTMONEYW | How did you get the money that you spent the last time you bought bath salts? | 1 = Legal employment  2 = Public assistance  3 = Partner or spouse  4 = Parents  5 = Other family member  6 = Drug sales  7 = Sex work  8 = Other illegal hustles  9 = Legal hustles or off-book work  10 = Savings  11 = Shelter or relief funds  13 = Other  97 = Don’t know  98 = Refused to answer | If NE 13 (Other) skip to |
| 089 | BSLTMONEYWO | What “other” source did you use to get the money that you spent the last time you bought bath salts? | Alpha Numeric Text Field |  |
| 090 | BSLTWHAT | The last time you bought bath salts, what were you buying? | 1 = MDPV  2 = Mephedrone (M-cat, meow, drone)  3 = Methylone (M1, Explosion)  4 = Butylone/ bk-MBDB  5= Other  97 = Don’t know  98 = Refused to answer | If NE 6 (Other) Skip to |
| 091 | BSLTWHATO | What “other” were you buying the last time you bought bath salts? | Alpha Numeric Text Field |  |
| 092 | BSLTEXP | Was the quality or experience what you expected the last time you used bath salts? | 0=No  1=Yes  97 = Don’t know  98 = Refused to answer | If NE 1(Yes) Skip to |
| 093 | BSLTEXPDIFF | The last time you used bath salts how was the quality or experience different from your expectations? | 1 = Too stimulating (nervousness, anxiety, anger)  2 = Too calming (fatigue, lethargy)  3 = Other  97 = Don’t know  98 = Refused to answer | If NE 3(Other) Skip to |
| 094 | BSLTEXPDIFFO | The last time you used bath salts, what was the “other” way your experience was different from your expectations? | Alpha Numeric Text Field |  |
| 095 | MOLLY | Have you ever, even once, used Molly? | 0=No  1=Yes  97 = Don’t know  98 = Refused to answer | If NE 1(Yes) Skip to |
| 096 | MOWHEN | How long has it been since you last used Molly? | 1=Within past 30 days  2=More than 30 days ago but within the past 12 months  3=More than 12 months ago  97=Don’t Know  98=Refused to Answer | If 3 (More than 12 months ago), skip to |
| 097 | PASTYRMODAYS | On average, how many days did you use Molly each month in the past 12 months? | F2.0  97=Don’t Know  98=Refused to Answer |  |
| 098 | PAST30MODAYS | Think specifically about the past 30 days, from [DATE] up to and including today. On how many of those days did you use Molly? | F2.0  97=Don’t Know  98=Refused to Answer |  |
| 099 | MOLEARN | How did you learn about Molly? | 1 = Relative  2 = Partner or lover  3 = Work associate  4 = Friend/Acquaintance  5 = Dealer  6 = Media  7 = Other  97 = Don’t know  98 = Refused to answer | If NE 7 (Other) Skip to |
| 100 | MOLEARNOTHER | What “other” way did you learn about Molly? | Alpha Numeric Text |  |
| 101 | MONUMFRIEND | How many of your friends would you estimate use Molly? | 1 = None  2 = A few  3 = Some  4 = Most  5 = All  97 = Don’t know  98 = Refused to answer |  |
| 102 | MOHOWPRIMARY | What is your primary way of using Molly? | 1=Smoke  2=Swallow  3=Snort  4=Inject  5=Other  97=Don’t Know  98=Refused to Answer | If NE 5 (Other) skip to |
| 103 | MOHOWOTHER | Explain the “other” way you use Molly | Alpha Numeric Text Field |  |
| 104 | MOHOWSECOND | What is your secondary way of using Molly? | 1=Smoke  2=Swallow  3=Snort  4=Inject  5=Other  6=No secondary method  97=Don’t Know  98=Refused to Answer | If NE 5 (Other) skip to |
| 105 | MOHOWOTHSEC | Explain the “other” secondary way you use Molly | Alpha Numeric Text Field |  |
| 106 | MOALCOHOL | Do you use Molly with alcohol? | 0=No  1=Yes  97=Don’t Know  98=Refused to Answer |  |
| 107 | MOOTHDRUG | Do you use Molly with other drugs? | 0=No  1=Yes  97=Don’t Know  98=Refused to Answer | If 0(No) skip to |
| 108 | MOOTHDRUGD | What other drugs do you use with Molly? | Alpha Numeric Text Field |  |
| 109 | MOWHERE | Where do you usually use Molly? | 1=Home, apartment, or dorm  2=Friend’s Home  3=Parties  4=Concerts/Festivals  5 = Clubs/Bars  6=Park, nature, outdoor  7=On the street, sidewalk  8=Other  97=Don’t Know  98=Refused to Answer | If NE 8(Other), skip to |
| 110 | MOWHEREOTHR | What other location do you typically use Molly? | Alpha Numeric Text Field |  |
| 111 | MOALONE | Do you usually use Molly by yourself or with other people? | 1=Alone  2=With Other People | If 1 (Alone) skip to |
| 112 | MOWITHWHO | Who do you usually use Molly with? | Alpha Numeric Text Field |  |
| 113 | MOWHY | What has been the most important reason for your using Molly? | 1=To Experiment – See What It’s Like  2=To Relax  3=To Feel Good or Get High  4=To increase sexual arousal and/or performance  5=To Have a Good Time with Friends  6=To Get Away from My Problems or Troubles  7=Because of Boredom, Nothing Else to Do  8=Because of Anger or Frustration  9=To Increase the Effect of Some Other Drug  10=To Decrease/Offset the Effects of Some Other Drug  11=Because I am “Hooked” – I Have to Have It  12=Other  97=Don’t Know  98=Refused to Answer | If NE 12(Other) skip to |
| 114 | MOWHYOTHER | What is the other primary reason for using Molly? | Alpha Numeric Text Field |  |
| For the next few questions I’m going to ask you whether you have had certain problems because of your use of Molly. | | | | |
| 115 | MOREGRET | Has Molly caused you to do things you later regretted? | 0=No  1=Yes  97=Don’t Know  98=Refused to Answer |  |
| 116 | MOHURTSCH | Has your use of Molly hurt your performance in school and/or on the job? | 0=No  1=Yes  97=Don’t Know  98=Refused to Answer |  |
| 117 | MOPOLICETROB | Has your use of Molly caused you to get in trouble with the police? | 0=No  1=Yes  97=Don’t Know  98=Refused to Answer |  |
| 118 | MOPROBOTHER | Has your use of Molly caused you other problems? | 0=No  1=Yes  97=Don’t Know  98=Refused to Answer | If 0 (No) Skip to |
| 119 | MOPROBOTHERO | What other problems has your use of Molly caused you? | Alpha Numeric Text Field. |  |
| 120 | MOALOT | During the past 12 months, was there a month or more when you spent a lot of your time getting or using Molly? | 0=No  1=Yes  97=Don’t Know  98=Refused to Answer |  |
| 121 | MOGETOVER | During the past 12 months, was there a month or more when you spent a lot of your time getting over the effects of the Molly you used? | 0=No  1=Yes  97=Don’t Know  98=Refused to Answer |  |
| 122 | MOEFFECTS | During the past 12 months, did you need to use more Molly than you used to in order to get the effect you wanted? | 0=No  1=Yes  97=Don’t Know  98=Refused to Answer |  |
| 123 | MOCTDWN | During the past 12 months, did you want to or try to cut down or stop using Molly? | 0=No  1=Yes  97=Don’t Know  98=Refused to Answer | If 0(No) skip to |
| 124 | MOCTDWNALO | During the past 12 months, did you cut down or stop using Molly at least one time? | 0=No  1=Yes  97=Don’t Know  98=Refused to Answer | If 0(No) skip to |
| 125 | MOSYMPTOMS | Please look at these symptoms. During the past 12 months, did you have any of these symptoms after you cut back or stopped using Molly?  *Feeling tired or exhausted  *Having bad dreams  *Having trouble sleeping or sleeping more than usual  *Feeling blue or depressed  *Feeling hungry more often  *Feeling either very slowed down or like you couldn’t sit still | 0=No  1=Yes  97=Don’t Know  98=Refused to Answer |  |
| 126 | MOWORSE | During the past 12 months, did you have any problems with your emotions, nerves, or mental health that were caused or made worse by using Molly? | 0=No  1=Yes  97=Don’t Know  98=Refused to Answer | If 0 (No) skip to |
| 127 | MOUSEANYWAY | Did you continue to use Molly even though you thought it was causing you to have problems with your emotions, nerves, or mental health? | 0=No  1=Yes  97=Don’t Know  98=Refused to Answer |  |
| 128 | MOHLTSPROB | During the past 12 months, did you have any health problems that were probably caused or made worse by your use of Molly? | 0=No  1=Yes  97=Don’t Know  98=Refused to Answer | If 0(No) skip to |
| 129 | MOHLTHPROBA | Did you continue to use Molly even though you thought it was causing you to have physical health problems? | 0=No  1=Yes  97=Don’t Know  98=Refused to Answer |  |
| 130 | MOPROBFAM | During the past 12 months, did you have any problems with family or friends that were probably caused by your use of Molly? | 0=No  1=Yes  97=Don’t Know  98=Refused to Answer | If 0(No) skip to |
| 131 | MOPROBFAMA | Did you continue to use Molly even though you thought it caused problems with family or friends? | 0=No  1=Yes  97=Don’t Know  98=Refused to Answer |  |
| 132 | MOFIGHT | Have you ever gotten into a fight or done anything violent after using Molly? | 0 = No  1 = Yes  97 = Don’t Know  98 = Refused to Answer |  |
| 133 | MOVICTIM | Have you ever been a victim of violence while using Molly? | 0 = No  1 = Yes  97 = Don’t Know  98 = Refused to Answer |  |
| 134 | MOVIOLENCE | Have you ever been a victim of violence while using Molly? | 0 = No  1 = Yes  97 = Don’t Know  98 = Refused to Answer |  |
| 135 | MOER | Have you ever gone to an emergency room after using Molly? | 0=No  1=Yes  97=Don’t Know  98=Refused to Answer |  |
| 136 | MOPOISONC | Have you ever called a poison control center after using Molly? | 0=No  1=Yes  97=Don’t Know  98=Refused to Answer |  |
| 137 | MOOBTAIN | Now think about the last time you used Molly. How did you get this Molly? | 1 = Bought it  2 = I traded something else for it  3 = I got it for free or shared someone else’s  4 = I made it myself  97 = Don’t know  98 = Refused to answer |  |
| 138 | MOFORM | What form of Molly did you acquire the last time you used Molly? | (other) (other)  3 = Capsules  4 = Pills or tablets  5 = Other  97 = Don’t Know  98 = Refused to answer | If NE 5 (Other) Skip to |
| 139 | MOFORMOTH | What “other” form of Molly did you use? | Alpha Numeric Text Field- for other |  |
| 140 | MOUNIT | What was the unit type of Molly that you received the last time you used Molly? | 1 = Small bag or sack  2 = Vials/small jars or bottles  3 = Grams  4 = Ounces  5 = Other  97 = Don’t Know  98 = Refused to answer | If NE 5 (Other) Skip to |
| 141 | MOUNITOTHER | What other unit type did you receive? | Alpha Numeric Text Field |  |
| 142 | MOGPRICE | In general, what does Molly cost? [Price in dollars] | F5.2 |  |
| 143 | MOGPRICEUNIT | What is the unit of the general price for Molly? | 1 = Small bag or sack  2 = Vials/small jars or bottles  3 = Grams  4 = Ounces  5 = Other  97 = Don’t Know  98 = Refused to answer | If NE 3 (Grams) or 4 (Ounces) Skip to |
| 144 | MOGPRICEAMT | How many grams/ounces is the general price for Molly? | F5.2 |  |
| 144b | MOGUNITOTH | What other unit type? | Alphanumeric text field |  |
| 145 | MOBUY | Have you ever bought Molly yourself? | 0 = No  1 = Yes  97 = Don’t know  98 = Refused to answer | If NE 1(Yes), Skip to |
| 146 | MOLTWHO | The last time you bought Molly who did you buy it from? | 1 = A friend/acquaintance  2 = A relative or family member  3 = Someone I had just met or didn’t know well  4 = The Internet  5 = Delivery service  6 = Dealer  7 = Other  97 = Don’t know  98 = Refused to answer | If NE 7 (Other) Skip to |
| 147 | MOLTWHOO | What “other” source did you buy Molly from? | Alpha Numeric Text Field |  |
| 148 | MOLTBUYWHERE | The last time you bought Molly, where were you when you bought it? | 1 = At a club, bar or festival  2 = At a school  3 = At a home, apartment, or dorm  4 = Outside in a public area (e.g., street or park)  5 = Other  97 = Don’t know  98 = Refused to answer | If NE 6 (Other) Skip to |
| 149 | MOLTBUYWHEREO | What “other” location were you in the last time you bought Molly? | Alpha Numeric Text Field |  |
| 150 | MOLTBUYRELATION | What is your relationship with your usual Molly source? | 1 = Relative  2 = Partner or lover  3 = Work associate  4 = Friend/Acquaintance  5 = Dealer (Not a friend/relative/partner)  6 = No personal relationship  97 = Don’t know  98 = Refused to answer |  |
| 151 | MOLTBUYUNIT | The last time you bought Molly, what units did you buy it in? | 1 = grams  2 = ounces  3 = bags  4 = vials  5 = other  97 = Don’t know  98 = Refused to answer | Else if NE 5 (Other) Skip to |
| 152 | MOLTBUYUNITO | The last time you bought Molly what “other” unit did you buy it in? | Alpha Numeric Text Field |  |
| 153 | MOLTBUYAMT | The last time you bought Molly, what number of gms/ozs/bags/vials/other did you buy? [Use the unit respondent provided in previous question] | F5.2 |  |
| 154 | MOLTMONEY | The last time you bought Molly, how much money did you spend? (whole dollars) | F4.0 |  |
| 155 | MOLTMONEYW | How did you get the money that you spent the last time you bought Molly? | 1 = Legal employment  2 = Public assistance  3 = Partner or spouse  4 = Parents  5 = Other family member  6 = Drug sales  7 = Sex work  8 = Oher illegal hustles  9 = Legal hustles or off-books work  10 = Savings  11 = Shelter or relief funds  13 = Other  97 = Don’t know  98 = Refused to answer | If NE 13 (Other) skip to |
| 156 | MOLTMONEYWO | What “other” source did you use to get the money that you spent the last time you bought Molly? | Alpha Numeric Text Field |  |
| 157 | MOLTWHAT | The last time you bought Molly, what were you buying? | 1 = MDMA  2 = Ecstasy  3 = MDA  4 = Other  97 = Don’t know  98 = Refused to answer | If NE 6 (Other) Skip to |
| 158 | MOLTWHATO | What “other” substance were you buying the last time you bought Molly? | Alpha Numeric Text Field |  |
| 159 | MOLTEXP | Was the quality or experience what you expected the last time you used Molly? | 0=No  1=Yes  97 = Don’t know  98 = Refused to answer | If NE 1(Yes) Skip to |
| 160 | MOLTEXPDIFF | The last time you used Molly how was the quality or experience different from your expectations? | 1 = Too stimulating (nervousness, anxiety, anger)  2 = Too calming (fatigue, lethargy)  3 = Other  97 = Don’t know  98 = Refused to answer | If NE 3(Other) Skip to |
| 161 | MOLTEXPDIFFO | The last time you used Molly, what was the “other” way your experience was different from your expectations? | Alpha Numeric Text Field |  |
| 162 | COCAINE | Have you ever, even once, used Cocaine? | 0=No  1=Yes  97 = Don’t Know  98 = Refused to Answer | If 0(No) skip to |
| 163 | COWHEN | How long has it been since you last used Cocaine? | 1=Within past 30 days  2=More than 30 days ago but within the past 12 months  3=More than 12 months ago  97=Don’t Know  98=Refused to Answer |  |
| 164 | PASTYRCODAYS | On average, how many days did you use Cocaine each month in the past 12 months? | F2.0  97=Don’t Know  98=Refused to Answer |  |
| 165 | PAST30CODAYS | Think specifically about the past 30 days, from [DATE] up to and including today. On how many of those days did you use Cocaine? | F2.0  97=Don’t Know  98=Refused to Answer |  |
| 166 | COHOW | How do you use Cocaine? | 1=Smoke  2=Swallow  3=Snort  4=Inject  5=Other  97=Don’t Know  98=Refused to Answer | If NE 5(Other) skip to |
| 167 | COHOWOTHER | Explain the “other” way you use Cocaine | Alpha Numeric Text Field |  |
| 168 | CRACK | Have you ever, even once, used Crack? | 0=No  1=Yes | If 0(No) skip to |
| 169 | CRWHEN | How long has it been since you last used Crack? | 1=Within past 30 days  2=More than 30 days ago but within the past 12 months  3=More than 12 months ago  97=Don’t Know  98=Refused to Answer |  |
| 170 | PASTYRCRDAYS | On average, how many days did you use Crack each month in the past 12 months? | F2.0  97=Don’t Know  98=Refused to Answer |  |
| 171 | PAST30CRDAYS | Think specifically about the past 30 days, from [DATE] up to and including today. On how many of those days did you use Crack? | F2.0  97=Don’t Know  98=Refused to Answer |  |
| 172 | CRLEARN | How did you learn about Crack? | 1 = Relative  2 = Partner or lover  3 = Work associate  4 = Friend/Acquaintance  5 = Dealer  6 = Media  7 = Other  97 = Don’t know  98 = Refused to answer | If NE 7 (Other) Skip to |
| 173 | CRLEARNOTHER | What “other” way did you learn about Crack? | Alpha Numeric Text Field |  |
| 174 | CRNUMFRIEND | How many of your friends would you estimate use Crack? | 1 = None  2 = A few  3 = Some  4 = Most  5 = All  97 = Don’t know  98 = Refused to answer |  |
| 175 | CRHOWPRIMARY | What is your primary way of using Crack? | 1=Smoke  2=Swallow  3=Snort  4=Inject  5=Other  97=Don’t Know  98=Refused to Answer | If NE 5 (Other), skip to |
| 176 | CRHOWOTHER | Explain the “other” way you use Crack | Alpha Numeric Text Field |  |
| 177 | CRHOWSECOND | What is your secondary way of using crack? | 1=Smoke  2=Swallow  3=Snort  4=Inject  5=Other  6=No secondary method  97=Don’t Know  98=Refused to Answer | If NE 5 (Other) skip to |
| 178 | CRHOWOTHSEC | Explain the “other” secondary way you use crack | Alpha Numeric Text Field |  |
| 179 | CRALCOHOL | Do you use Crack with alcohol? | 0=No  1=Yes  97=Don’t Know  98=Refused to Answer |  |
| 180 | CROTHDRUG | Do you use Crack with other drugs? | 0=No  1=Yes  97=Don’t Know  98=Refused to Answer | If 0(No) skip to |
| 181 | CROTHDRUGD | What other drugs do you use with Crack? | Alpha Numeric Text Field |  |
| 182 | CRWHERE | Where do you usually use Crack? | 1=Home, apartment, dorm  2=Friend’s Home  3=Parties  4=Concerts  5=Clubs/Bars  5=Park, nature, outdoor  6=On the street, sidewalk  7=Other  97=Don’t Know  98=Refused to Answer | If NE 7(Other), skip to |
| 183 | CRWHEREOTHR | In what “other” location do you typically use Crack? | Alpha Numeric Text Field |  |
| 184 | CRALONE | Do you usually use Crack by yourself or with other people? | 1=Alone  2=With Other People  97=Don’t Know  98=Refused to Answer | If 1 (Alone) skip to |
| 185 | CRWITHWHO | Who do you usually use Crack with? | Alpha Numeric Text Field |  |
| 186 | CRWHY | What has been your primary reason for using Crack? | 1=To Experiment – See What It’s Like  2=To Relax  3=To Feel Good or Get High  4=To increase sexual arousal and/or performance  5=To Have A Good Time with Friends  6=To Get Away From My Problems or Troubles  7=Because of Boredom, Nothing Else to Do  8=Because of Anger or Frustration  9=To Increase the Effect of Some Other Drug  10=To Decrease/Offset the Effects of Some Other Drug  11=Because I am “Hooked” – I Have to Have It  12=Other  97=Don’t Know  98=Refused to Answer | If NE 12(Other) skip to |
| 187 | CRWHYOTHER | What is the “other” primary reason for using Crack? | Alpha Numeric Text Field |  |
| For the next few questions I’m going to ask you whether you have had certain problems because of your use of crack. | | | | |
| 188 | CRREGRET | Has cracks caused you to do things you later regretted? | 0=No  1=Yes  97=Don’t Know  98=Refused to Answer |  |
| 189 | CRHURTSCH | Has your use of crack hurt your performance in school and/or on the job? | 0=No  1=Yes  97=Don’t Know  98=Refused to Answer |  |
| 190 | CRPOLICETROB | Has your use of crack caused you to get in trouble with the police? | 0=No  1=Yes  97=Don’t Know  98=Refused to Answer |  |
| 191 | CRPROBOTHER | Has your use of crack caused you other problems? | 0=No  1=Yes  97=Don’t Know  98=Refused to Answer | If 0 (No), skip to |
| 192 | CRPROBOTHERO | What other problems has your use of crack caused you? | Alpha Numeric Text Field |  |
| 193 | CRALOT | During the past 12 months, was there a month or more when you spent a lot of your time getting or using Crack? | 0=No  1=Yes  97=Don’t Know  98=Refused to Answer |  |
| 194 | CRGETOVER | During the past 12 months, was there a month or more when you spent a lot of your time getting over the effects of the Crack you used? | 0=No  1=Yes  97=Don’t Know  98=Refused to Answer |  |
| 195 | CREFFECTS | During the past 12 months, did you need to use more crack than you used to in order to get the effect you wanted? | 0=No  1=Yes  97=Don’t Know  98=Refused to Answer |  |
|  |  |  |  |  |
| 196 | CRPCTDWN | During the past 12 months, did you want to or try to cut down or stop using Crack? | 0=No  1=Yes  97=Don’t Know  98=Refused to Answer | If 0(No) skip to |
| 197 | CRCTDWNALO | During the past 12 months, did you cut down or stop using Crack at least one time? | 0=No  1=Yes  97=Don’t Know  98=Refused to Answer | If 0(No) skip to |
| 198 | CRSYMPTOMS | Please look at these symptoms. During the past 12 months, did you have any of these symptoms after you cut back or stopped using Crack?  *Feeling tired or exhausted  *Having bad dreams  *Having trouble sleeping or sleeping more than usual  * Feeling blue or depressed  *Feeling hungry more often  *Feeling either very slowed down or like you couldn’t sit still | 0=No  1=Yes  97=Don’t Know  98=Refused to Answer |  |
| 199 | CRWORSE | During the past 12 months, did you have any problems with your emotions, nerves, or mental health that were caused or made worse by using Crack? | 0=No  1=Yes  97=Don’t Know  98=Refused to Answer | If 0(No) skip to |
| 200 | CRUSEANYWAY | Did you continue to use Crack even though you thought it was causing you to have problems with your emotions, nerves, or mental health? | 0=No  1=Yes  97=Don’t Know  98=Refused to Answer |  |
| 201 | CRHLTSPROB | During the past 12 months, did you have any health problems that were probably caused or made worse by your use of Crack? | 0=No  1=Yes  97=Don’t Know  98=Refused to Answer | If 0(No) skip to |
| 202 | CRHLTHPROBA | Did you continue to use Crack even though you thought it was causing you to have physical health problems? | 0=No  1=Yes  97=Don’t Know  98=Refused to Answer |  |
| 203 | CRPROBFAM | During the past 12 months, did you have any problems with family or friends that were probably caused by your use of Crack? | 0=No  1=Yes  97=Don’t Know  98=Refused to Answer | If 0(No) skip to |
| 204 | CRPROBFAMA | Did you continue to use Crack even though you thought it caused problems with family or friends? | 0=No  1=Yes  97=Don’t Know  98=Refused to Answer |  |
| 205 | CRFIGHT | Have you ever gotten into a fight or done anything violent after using crack? | 0 = No  1 = Yes  97 = Don’t Know  98 = Refused to Answer |  |
| 206 | CRVICTIM | Have you ever been a victim of violence while using crack? | 0 = No  1 = Yes  97 = Don’t Know  98 = Refused to Answer |  |
| 207 | CRVIOLENCE | Have you ever seen anyone become violent while using crack? | 0 = No  1 = Yes  97 = Don’t Know  98 = Refused to Answer |  |
| 208 | CRER | Have you ever gone to an emergency room after using crack? | 0=No  1=Yes  97=Don’t Know  98=Refused to Answer |  |
| 209 | CRPOISONC | Have you ever called a poison control center after using crack? | 0=No  1=Yes  97=Don’t Know  98=Refused to Answer |  |
| 210 | CROBTAIN | Now think about the last time you used crack. How did you get it? | 1 = Bought it  2 = I traded something else for it  3 = I got it for free or shared someone else’s  4 = I made it myself  5 = Other  97 = Don’t know  98 = Refused to answer | If NE 5 (Other) skip to |
|  | CROBTAINO | What other way did you get crack? | F5.2 |  |
| 211 | CRGPRICE | In general, what does crack cost? [Price in dollars] | F5.2 |  |
| 212 | CRGPRICEUNIT | What is the unit of the general price for crack? | 1 = Small bag or sack  2 = Vials/small jars or bottles  3 = Grams  4 = Ounces  5 = Other  97 = Don’t Know  98 = Refused to answer | If NE 3 (Grams) or 4 (Ounces) Skip to |
| 212b | CRGUNITOTH | What “other” unit type? | Text Field |  |
| 213 | CRGPRICEAMT | How many grams/ounces is the general price for crack? | F5.2 |  |
| 214 | CRBUY | Have you ever bought crack yourself? | 0 = No  1 = Yes  97 = Don’t know  98 = Refused to answer | If NE 1(Yes), Skip to |
| 215 | CRLTWHO | The last time you bought crack who did you buy it from? | 1 = A friend/acquaintance  2 = A relative or family member  3 = Someone I had just met or didn’t know well  4 = The Internet  5 = Delivery service  6= Dealer  7 = Other  97 = Don’t know  98 = Refused to answer | If NE 7 (Other) Skip to |
| 216 | CRLTWHOO | What “other” source did you buy crack from? | Alpha Numeric Text Field |  |
| 217 | CRLTBUYWHERE | The last time you bought crack, where were you when you bought it? | 1 = At a club, bar or festival  2 = At a school 3 = At a home, apartment, or dorm  4 = Outside in a public area (e.g., street or park)  5 = Other  97 = Don’t know  98 = Refused to answer | If NE 6 (Other) Skip to |
| 218 | CRLTBUYWHEREO | What “other” location were you in the last time you bought crack? | Alpha Numeric Text Field |  |
| 219 | CRLTBUYRELATION | What is your relationship with your usual crack source? | 1 = Relative  2 = Partner or lover  3 = Work associate  4 = Friend/Acquaintance  5 = Dealer (not relative/friend/partner)  6 = No personal relationship  97 = Don’t know  98 = Refused to answer |  |
| 220 | CRLTBUYUNIT | The last time you bought crack, what units did you buy it in? | 1 = grams  2 = ounces  3 = bags  4 = vials  5 = other  97 = Don’t know  98 = Refused to answer | Else if NE 5 (Other) Skip to |
| 221 | CRLTBUYUNITO | The last time you bought crack what “other” unit did you buy it in? | Alpha Numeric Text Field |  |
| 222 | CRLTBUYAMT | The last time you bought crack, what number of gms/ozs/bags/vials/other did you buy? (Use the unit from respondent’s previous answer) | F5.2 |  |
| 223 | CRLTMONEY | The last time you bought crack, how much money did you spend? (whole dollars) | F4.0 |  |
| 22 | CRLTMONEYW | How did you get the money that you spent the last time you bought crack? | 1 = Legal employment  2 = Public assistance  3 = Partner or spouse  4 = Parents  5 = Other family member  6 = Drug sales  7 = Sex work  8 = Oher illegal hustles  9 = Legal hustles or off-book work  10 = Savings  11 = Shelter or relief funds  13 = Other  97 = Don’t know  98 = Refused to answer | If NE 13 (Other) skip to |
| 225 | CRLTMONEYWO | What “other” source did you use to get the money that you spent the last time you bought crack? | Alpha Numeric Text Field |  |
| 226 | CRLTEXP | Was the quality or experience what you expected the last time you used crack? | 0=No  1=Yes  97 = Don’t know  98 = Refused to answer | If NE 1(Yes) Skip to |
| 227 | CRLTEXPDIFF | The last time you used crack how was the quality or experience different from your expectations? | 1 = Too stimulating (nervousness, anxiety, anger)  2 = Too calming (fatigue, lethargy)  3 = Other  97 = Don’t know  98 = Refused to answer | If NE 3(Other) Skip to |
| 228 | CRLTEXPDIFFO | The last time you used crack, what was the “other” way your experience was different from your expectations? | Alpha Numeric Text Field |  |
| 229 | METH | Have you ever, even once, used Methamphetamine? | 0=No  1=Yes | If 0(No) skip to |
| 230 | MTWHEN | How long has it been since you last used Methamphetamine? | 1=Within past 30 days  2=More than 30 days ago but within the past 12 months  3=More than 12 months ago  97=Don’t Know  98=Refused to Answer |  |
| 231 | PASTYRMTDAYS | On average, how many days did you use Methamphetamine each month in the past 12 months? | F2.0  97=Don’t Know  98=Refused to Answer |  |
| 232 | PAST30MTDAYS | Think specifically about the past 30 days, from [DATE] up to and including today. On how many of those days did you use Methamphetamine? | F2.0  97=Don’t Know  98=Refused to Answer |  |
| 233 | MTLEARN | How did you learn about Methamphetamine? | 1 = Relative  2 = Partner or lover  3 = Work associate  4 = Friend/Acquaintance  5 = Dealer  6 = Media  7 = Other  97 = Don’t know  98 = Refused to answer | If NE 7 (Other) Skip to |
| 234 | MTLEARNOTHER | What “other” way did you learn about Methamphetamine? | Alpha numeric text field |  |
| 235 | MTNUMFRIEND | How many of your friends would you estimate use Methamphetamine? | 1 = None  2 = A few  3 = Some  4 = Most  5 = All  97 = Don’t know  98 = Refused to answer |  |
| 236 | MTHOWPRIMARY | What is your primary way of using Methamphetamine? | 1=Smoke  2=Swallow  3=Snort  4=Inject  5=Other  97=Don’t Know  98=Refused to Answer | If NE 5 (Other) skip to |
| 237 | MTHOWOTHER | Explain the “other” way you use Methamphetamine | Alpha Numeric Text Field |  |
| 238 | MTHOWSECOND | What is your secondary way of using methamphetamine? | 1=Smoke  2=Swallow  3=Snort  4=Inject  5=Other  6=No secondary method  97=Don’t Know  98=Refused to Answer | If NE 5 (Other) skip to |
| 239 | MTHOWOTHSEC | Explain the “other” secondary way you use methamphetamine | Alpha Numeric Text Field |  |
| 240 | MTALCOHOL | Do you use Methamphetamine with alcohol? | 0=No  1=Yes  97=Don’t Know  98=Refused to Answer |  |
| 241 | MTOTHDRUG | Do you use Methamphetamine with other drugs? | 0=No  1=Yes  97=Don’t Know  98=Refused to Answer | If 0(No) skip to |
| 242 | MTOTHDRUGD | What other drugs do you use with Methamphetamine? | Alpha Numeric Text Field |  |
| 243 | MTWHERE | Where do you usually use Methamphetamine? | 1=Home, apartment, or dorm  2=Friend’s Home  3=Parties  4=Concerts/Festivals  5 = Clubs/Bars  6=Park, nature, outdoor  7=On the street, sidewalk  8=Other  97=Don’t Know  98=Refused to Answer | If NE 7(Other), skip to |
| 244 | MTWHEREOTHR | In what “other” location do you typically use Methamphetamine? | Alpha Numeric Text Field |  |
| 245 | MTALONE | Do you usually use Methamphetamine by yourself or with other people? | 1=Alone  2=With Other People | If 1 (Alone) skip to |
| 246 | MTWITHWHO | Who do you usually use Methamphetamine with? | Alpha Numeric Text Field |  |
| 247 | MTWHY | What has been your primary reason for using Methamphetamine? | 1=To Experiment – See What It’s Like  2=To Relax  3=To Feel Good or Get High  4=To increase sexual arousal or performance  5=To Have a Good Time with Friends  6=To Get Away from My Problems or Troubles  7=Because of Boredom, Nothing Else to Do  8=Because of Anger or Frustration  9=To Increase the Effect of Some Other Drug  10=To Decrease/Offset the Effects of Some Other Drug  11=Because I am “Hooked” – I Have to Have It  12=Other  97=Don’t Know  98=Refused to Answer | If NE 12(Other) skip to |
| 248 | MTWHYOTHER | What is your “other” primary reason for using Methamphetamine? | Alpha Numeric Text Field |  |
| For the next few questions I’m going to ask you whether you have had certain problems because of your use of methamphetamine. | | | | |
| 249 | MTREGRET | Has methamphetamine caused you to do things you later regretted? | 0=No  1=Yes  97=Don’t Know  98=Refused to Answer |  |
| 250 | MTHURTSCH | Has your use of methamphetamine hurt your performance in school and/or on the job? | 0=No  1=Yes  97=Don’t Know  98=Refused to Answer |  |
| 251 | MTPOLICETROB | Has your use of methamphetamine caused you to get in trouble with the police? | 0=No  1=Yes  97=Don’t Know  98=Refused to Answer |  |
| 252 | MTPROBOTHER | Has your use of methamphetamine caused you other problems? | 0=No  1=Yes  97=Don’t Know  98=Refused to Answer | If 0 (No), skip to |
| 253 | MTPROBOTHERO | What other problems has your use of methamphetamine caused you? | Alpha Numeric Text Field |  |
| 254 | MTALOT | During the past 12 months, was there a month or more when you spent a lot of your time getting or using Methamphetamine? | 0=No  1=Yes  97=Don’t Know  98=Refused to Answer |  |
| 255 | MTGETOVER | During the past 12 months, was there a month or more when you spent a lot of your time getting over the effects of the Methamphetamine you used? | 0=No  1=Yes  97=Don’t Know  98=Refused to Answer |  |
| 256 | MTEFFECTS | During the past 12 months, did you need to use more methamphetamine than you used to in order to get the effect you wanted? | 0=No  1=Yes  97=Don’t Know  98=Refused to Answer |  |
| 257 | MTPCTDWN | During the past 12 months, did you want to or try to cut down or stop using Methamphetamine? | 0=No  1=Yes  97=Don’t Know  98=Refused to Answer | If 0(No) skip to |
| 258 | MTCTDWNALO | During the past 12 months, did you cut down or stop using Methamphetamine at least one time? | 0=No  1=Yes  97=Don’t Know  98=Refused to Answer | If 0(No) skip to |
| 259 | MTSYMPTOMS | Please look at these symptoms. During the past 12 months, did you have any of these symptoms after you cut back or stopped using Methamphetamine?  *Feeling tired or exhausted  *Having bad dreams  *Having trouble sleeping or sleeping more than usual  *Feeling blue or depressed  *Feeling hungry more often  *Feeling either very slowed down or like you couldn’t sit still | 0=No  1=Yes  97=Don’t Know  98=Refused to Answer |  |
| 260 | MTWORSE | During the past 12 months, did you have any problems with your emotions, nerves, or mental health that were caused or made worse by using Methamphetamine? | 0=No  1=Yes  97=Don’t Know  98=Refused to Answer | If 0(No) skip to |
| 261 | MTUSEANYWAY | Did you continue to use Methamphetamine even though you thought it was causing you to have problems with your emotions, nerves, or mental health? | 0=No  1=Yes  97=Don’t Know  98=Refused to Answer |  |
| 262 | MTHLTSPROB | During the past 12 months, did you have any health problems that were probably caused or made worse by your use of Methamphetamine? | 0=No  1=Yes  97=Don’t Know  98=Refused to Answer | If 0(No) skip to |
| 263 | MTHLTHPROBA | Did you continue to use Methamphetamine even though you thought it was causing you to have physical health problems? | 0=No  1=Yes  97=Don’t Know  98=Refused to Answer |  |
| 264 | MTPROBFAM | During the past 12 months, did you have any problems with family or friends that were probably caused by your use of Methamphetamine? | 0=No  1=Yes  97=Don’t Know  98=Refused to Answer | If 0(No) skip to |
| 265 | MTPROBFAMA | Did you continue to use Methamphetamine even though you thought it caused problems with family or friends? | 0=No  1=Yes  97=Don’t Know  98=Refused to Answer |  |
| 266 | MTFIGHT | Have you ever gotten into a fight or done anything violent after using Methamphetamine? | 0 = No  1 = Yes  97 = Don’t Know  98 = Refused to Answer |  |
| 267 | MTVICTIM | Have you ever been a victim of violence while using Methamphetamine? | 0 = No  1 = Yes  97 = Don’t Know  98 = Refused to Answer |  |
| 268 | MTVIOLENCE | Have you ever seen anyone become violent while using Methamphetamine? | 0 = No  1 = Yes  97 = Don’t Know  98 = Refused to Answer |  |
| 269 | MTER | Have you ever gone to an emergency room after using Methamphetamine? | 0=No  1=Yes  97=Don’t Know  98=Refused to Answer |  |
| 270 | MTPOISONC | Have you ever called a poison control center after using Methamphetamine? | 0=No  1=Yes  97=Don’t Know  98=Refused to Answer |  |
| 271 | MTOBTAIN | Now think about the last time you used Methamphetamine. How did you get it? | 1 = Bought it  2 = I traded something else for it  3 = I got it for free or shared someone else’s  4 = I made it myself  97 = Don’t know  98 = Refused to answer |  |
| 271b | MTOBTAINOTH | What other way did you get Methamphetamine? | Text field |  |
| 272 | MTFORM | What form of Methamphetamine did you acquire the last time you used Methamphetamine? | 1 = Powder  2 = Crystals  3 = Capsules  4 = Pills or tablets  5 = Chunks  6 = Other  97 = Don’t know  98 = Refused to answer | If NE 6 (Other) skip to |
| 273 | MTFORMOTH | What “other” form of Methamphetamine did you use? | Alpha Numeric Text Field |  |
| 274 | MTUNIT | What was the unit type of methamphetamine that you received the last time you used it? | 1 = Small bag or sack  2 = Vials/small jars or bottles  3 = Grams  4 = Ounces  5 = Other  97 = Don’t Know  98 = Refused to answer | If NE 5 (Other), skip to |
| 275 | MTUNITOTHER | What “other” unit type did you receive? | Alpha Numeric Text Field |  |
| 276 | MTGPRICE | In general, what does Methamphetamine cost? [Price in dollars] | F5.2 |  |
| 277 | MTGPRICEUNIT | What is the unit of the general price for Methamphetamine? | 1 = Small bag or sack  2 = Vials/small jars or bottles  3 = Grams  4 = Ounces  5 = Other  97 = Don’t Know  98 = Refused to answer | If NE 3 (Grams) or 4 (Ounces) Skip to |
| 277b | MTGUNITOTH | What other unit type? | Alphanumeric text field |  |
| 278 | MTGPRICEAMT | How many grams/ounces is the general price for Methamphetamine? | F5.2 |  |
| 279 | MTBUY | Have you ever bought Methamphetamine yourself? | 0 = No  1 = Yes  97 = Don’t know  98 = Refused to answer | If NE 1(Yes), Skip to |
| 280 | MTLTWHO | The last time you bought Methamphetamine who did you buy it from? | 1 = A friend/acquaintance  2 = A relative or family member  3 = Someone I had just met or didn’t know well  4 = The Internet  5 = Delivery service  6 = Dealer  7 = Other  97 = Don’t know  98 = Refused to answer | If NE 7 (Other) Skip to |
| 281 | MTLTWHOO | What “other” source did you buy Methamphetamine from? | Alpha Numeric Text Field |  |
| 282 | MTLTBUYWHERE | The last time you bought Methamphetamine, where were you when you bought it? | 1 = At a club, bar or festival  2 = At a school  3 = At a home, apartment, or dorm  4 = Outside in a public area (e.g., street or park)  5 = Other  97 = Don’t know  98 = Refused to answer | If NE 6 (Other) Skip to |
| 283 | MTLTBUYWHEREO | What “other” location were you in the last time you bought Methamphetamine? | Alpha Numeric Text Field |  |
| 284 | MTLTBUYRELATION | What is your relationship with your usual Methamphetamine source? | 1 = Relative  2 = Partner or lover  3 = Work associate  4 = Friend/acquaintance  5 = Dealer (not relative/friend/partner)  5 = No personal relationship  97 = Don’t know  98 = Refused to answer |  |
| 285 | MTLTBUYUNIT | The last time you bought Methamphetamine, what units did you buy it in? | 1 = grams  2 = ounces  3 = bags  4 = vials  5 = other  97 = Don’t know  98 = Refused to answer | If 6 (Did not buy Meth) Skip to  Else if NE 5 (Other) Skip to |
| 286 | MTLTBUYUNITO | The last time you bought Methamphetamine what other unit did you buy it in? | Alpha Numeric Text Field |  |
| 287 | MTLTBUYAMT | The last time you bought Methamphetamine, what number of gms/ozs/bags/vials/other [368] did you buy? | F5.2 |  |
| 288 | MTLTMONEY | The last time you bought Methamphetamine, how much money did you spend? (whole dollars) | F4.0 |  |
| 289 | MTLTMONEYW | How did you get the money that you spent the last time you bought Methamphetamine? | 1 = Legal employment  2 = Public assistance  3 = Partner or spouse  4 = Parents  5 = Other family member  6 = Drug sales  7 = Sex work  8 = Oher illegal hustles  9 = Legal hustles or off-book work  10 = Savings  11 = Shelter or relief funds  13 = Other  97 = Don’t know  98 = Refused to answer | If NE 13 (Other) skip to |
| 290 | MTLTMONEYWO | What “other” source did you use to get the money that you spent the last time you bought Methamphetamine? | Alpha Numeric Text Field |  |
| 291 | MTLTWHAT | The last time you bought Methamphetamine, what were you buying? | 1 = Methedrine  2 = Desoxyn  3 = Ephedrine  4 = Other  97 = Don’t know  98 = Refused to answer | If NE 4 (Other) Skip to |
| 292 | MTLTWHATO | What “other” substance were you buying the last time you bought Methamphetamine? | Alpha Numeric Text Field |  |
| 293 | MTLTEXP | Was the quality or experience what you expected the last time you used Methamphetamine? | 0=No  1=Yes  97 = Don’t know  98 = Refused to answer | If NE 1(Yes) Skip to |
| 294 | MTLTEXPDIFF | The last time you used Methamphetamine how was the quality or experience different from your expectations? | 1 = Too stimulating (nervousness, anxiety, anger)  2 = Too calming (fatigue, lethargy)  3 = Other  97 = Don’t know  98 = Refused to answer | If NE 3(Other) Skip to |
| 295 | MTLTEXPDIFFO | The last time you used Methamphetamine, what was the “other” way your experience was different from your expectations? | Alpha Numeric Text Field |  |
| 296 | TOBSMK | Have you ever smoked a cigarette? | 0 = No  1 = Yes  97 = Don’t Know  98 = Refused to Answer | If 0 (No) Skip to |
| 297 | TOBSMK100 | Have you smoked at least 100 cigarettes in your entire life? | 0 = No  1 = Yes  97 = Don’t Know  98 = Refused to Answer | If 0 (No) Skip to |
| 298 | TOBSMKDays | Do you smoke cigarettes every day, most days, or some days? | 1 = Some days  2 = Most days  3 = Every day  4 = Don’t smoke now |  |
| 299 | TOBSMK30days | In the past 30 days, how many cigarettes did you smoke per day, on average? | F3.0 |  |
| One drink is equivalent to a 12-ounce beer, a 5 ounce glass of wine, or a drink with one shot of liquor. | | | | |
| 300 | ETOH30DAYS | During the past 30 days, have you had at least one drink of any alcoholic beverage such as beer, wine, a malt beverage, or liquor? | 0 = No  1 = Yes  97 = Don’t Know  98 = Refused to Answer | If 0 (No) Skip to |
| 301 | ETOHDAYS | During the past 30 days, on how many days did you have at least one drink of any alcoholic beverage? | F2.0 |  |
| 302 | ETOHDRINKS | During the past 30 days, on the days when you drank, about how many drinks did you have on average? | F2.0 |  |
| 303 | ETOHBINGE | During the past 30 days, on how many days did you have 5 or more drinks on the same occasion? By ‘occasion’ we mean at the same time or within a couple of hours of each other. | F2.0 |  |
| 304 | ETOHMOST | During the past 30 days, what is the largest number of drinks you had on any occasion? | F3.0 |  |
| The next questions are about marijuana which is also called pot or weed. Marijuana is usually smoked, either in cigarettes, called joints, or in a pipe. It is sometimes cooked in food. | | | | |
| 305 | MARIJUANA | Have you ever, even once, used marijuana? | 0 = No  1 = Yes  97 = Don’t Know  98 = Refused to Answer | If 0 (No) Skip to |
| 306 | MARIJUANALAST | How long has it been since you last used marijuana? | 1 =- Within the past 30 days  2 = More than 30 days ago but within the past 12 months  3 = More than 12 months ago  97 = Don’t Know  98 = Refused to Answer | If 3 (More than 12 months ago) Skip to |
| 307 | MARIJUANADAYS | On average, how many days did you use marijuana each month during the past 12 months? | F2.0 |  |
| 308 | MARIJUANA30DAYS | Think specifically about the past 30 days up to and including today. On how many days did you use marijuana? | F2.0 |  |
| 309 | BLUNT | Have you ever smoked part or all of a cigar with marijuana in it – a blunt? | 0 = No  1 = Yes  97 = Don’t Know  98 = Refused to Answer | If 0 (No) Skip to |
| 310 | BLUNTLAST | How long has it been since you last smoked part or all of a blunt? | 1 =- Within the past 30 days  2 = More than 30 days ago but within the past 12 months  3 = More than 12 months ago  97 = Don’t Know  98 = Refused to Answer | If 3 (More than 12 months ago) Skip to |
| 311 | BLUNTDAYS | On average, how many days did you smoke part or all of a blunt each month during the past 12 months? | F2.0 |  |
| 312 | BLUNT30DAYS | Think specifically about the past 30 days up to and including today. On how many of those days did you smoke part or all of a blunt? | F2.0 |  |
| 313 | HEROIN | Have you, even once, used heroin? | 0 = No  1 = Yes  97 = Don’t Know  98 = Refused to Answer | If 0 (No) Skip to |
| 314 | HNLAST | How long has it been since you last used heroin? | 1 =- Within the past 30 days  2 = More than 30 days ago but within the past 12 months  3 = More than 12 months ago  97 = Don’t Know  98 = Refused to Answer | If 3 (More than 12 months ago) Skip to |
| 315 | HNDAYS | During the past 12 months, how many days on average did you use heroin EACH month? | F2.0 |  |
| 316 | HN30DAYS | Think specifically about the past 30 days up to and including today. On how many of those days did you use heroin? | F2.0 |  |
| 317 | PDRUGS | Have you ever, even once, used any prescription drugs non-medically? | 0 = No  1 = Yes  97 = Don’t Know  98 = Refused to Answer | If 0 (No) Skip to |
| 318 | POPIODS | Have you used prescription opioids non-medically? (Vicodin, Oxycontin, Percocet, etc.) | 0 = No  1 = Yes  97 = Don’t Know  98 = Refused to Answer |  |
| 319 | PBENZO | Have you used prescription benzodiazepines non-medically? (Xanax, Klonopin, Librium, Valium, etc.) | 0 = No  1 = Yes  97 = Don’t Know  98 = Refused to Answer |  |
| 320 | PSTIMULANT | Have you used prescription stimulants non-medically? (Adderall, Ritalin, Concerta, etc.) | 0 = No  1 = Yes  97 = Don’t Know  98 = Refused to Answer |  |
| 321 | POTHER | Have you used other prescription drugs non-medically? (please name) | Alpha Numeric Text Field  Specify, if yes |  |
| 321b | POTHERWHICH | What other prescription drugs have you used non-medically? | Text Field |  |
| 322 | PLAST | How long has it been since you last used prescription drugs non-medically? | 1 =- Within the past 30 days  2 = More than 30 days ago but within the past 12 months  3 = More than 12 months ago  97 = Don’t Know  98 = Refused to Answer | If 3 (More than 12 months ago) Skip to |
| 323 | PDAYS | On average, how many days did you use prescription drugs non-medically each month during the past 12 months? | F2.0 |  |
| 324 | P30DAYS | Think specifically about the past 30 days up to and including today. On how many of those days did you use prescription drugs non-medically? | F2.0 |  |
| 325 | SCANNABIS | Have you ever used synthetic weed/cannabis (Spice, K2, Mojo)? | 0 = No  1 = Yes  97 = Don’t Know  98 = Refused to Answer | If 0 (No) Skip to |
| 326 | SCLAST | How long has it been since you last used synthetic weed/cannabis? | 1 =- Within the past 30 days  2 = More than 30 days ago but within the past 12 months  3 = More than 12 months ago  97 = Don’t Know  98 = Refused to Answer | If 3 (More than 12 months ago) Skip to |
| 327 | SCDAYS | On average, how many days did you use synthetic weed/cannabis each month during the past 12 months? | F2.0 |  |
| 328 | SC30DAYS | Think specifically about the past 30 days up to and including today. On how many of those days did you use synthetic weed/cannabis? | F2.0 |  |
| 329 | SCLEARN | How did you learn about synthetic weed/cannabis? | 1 = Relative  2 = Partner or lover  3 = Work associate  4 = Friend/Acquaintance  5 = Dealer  6 = Media  7 = Other  97 = Don’t know  98 = Refused to answer | If NE 7 (Other) Skip to |
| 330 | SCLEARNOTHER | What other way did you learn about synthetic weed/cannabis? | Alpha Numeric Text Field |  |
| 331 | SCNUMFRIEND | How many of your friends would you estimate use synthetic weed/cannabis? | 1 = None  2 = A few  3 = Some  4 = Most  5 = All  97 = Don’t know  98 = Refused to answer |  |
| 332 | SCHOWPRIMARY | What is your primary way of using synthetic weed/cannabis? | 1=Smoke  2=Swallow  3=Snort  4=Inject  5=Other  97=Don’t Know  98=Refused to Answer | If NE 5 (Other) skip to |
| 333 | SCHOWOTHSEC | Explain the “other” secondary way you use synthetic weed/cannabis | Alpha Numeric Text Field |  |
| 334 | SCHOWSECOND | What is your secondary way of using synthetic weed/cannabis? | 1=Smoke  2=Swallow  3=Snort  4=Inject  5=Other  6=No secondary method  97=Don’t Know  98=Refused to Answer | If NE 5(Other) skip to |
| 335 | SCHOWOTHER | Explain the “other” way you use synthetic weed/cannabis | Alpha Numeric Text Field |  |
| 336 | SCALCOHOL | Do you use synthetic weed/cannabis with alcohol? | 0=No  1=Yes  97=Don’t Know  98=Refused to Answer |  |
| 337 | SCOTHDRUG | Do you use synthetic weed/cannabis with other drugs? | 0=No  1=Yes  97=Don’t Know  98=Refused to Answer | If 0 (No) skip to |
| 338 | SCOTHDRUGD | What other drugs do you use with synthetic weed/cannabis? | Alpha Numeric Text Field |  |
| 339 | SCWHERE | Where do you usually use synthetic weed/cannabis? | 1=Home, apartment, or dorm  2=Friend’s Home  3=Parties  4=Concerts/Festivals  5=Clubs/Bars  6=Park, nature, outdoor  7=On the street, sidewalk  8=Other  97=Don’t Know  98=Refused to Answer | If NE 8(Other), skip to |
| 340 | SCWHEREOTHR | In what “other” location do you typically use synthetic weed/cannabis? | Alpha Numeric Text Field |  |
| 341 | SCALONE | Do you usually use synthetic weed/cannabis by yourself or with other people? | 1=Alone  2=With Other People | If 1 (Alone) skip to |
| 342 | SCWITHWHO | Who do you usually use synthetic weed/cannabis with? | Alpha Numeric Text Field |  |
| 343 | SCWHY | What has been your primary reason for using synthetic weed/cannabis? | 1=To Experiment – See What It’s Like  2=To Relax  3=To Feel Good or Get High  4=To increase sexual arousal or performance  5=To Have a Good Timewith Friends  6=To Get Away from My Problems or Troubles  7=Because of Boredom, Nothing Else to Do  8=Because of Anger or Frustration  9=To Increase the Effect of Some Other Drug  10=To Decrease/Offset the Effects of Some Other Drug  11=Because I am “Hooked” – I Have to Have It  12=Other  97=Don’t Know  98=Refused to Answer | If NE 12(Other) skip to |
| 344 | SCWHYOTHER | What is your “other” primary reason for using synthetic weed/cannabis? | Alpha Numeric Text Field |  |
| For the next few questions I’m going to ask you whether you have had certain problems because of your use of synthetic weed/cannabis. | | | | |
| 345 | SCREGRET | Has synthetic weed/cannabis caused you to do things you later regretted? | 0=No  1=Yes  97=Don’t Know  98=Refused to Answer |  |
| 346 | SCHURTSCH | Has your use of synthetic weed/cannabis hurt your performance in school and/or on the job? | 0=No  1=Yes  97=Don’t Know  98=Refused to Answer |  |
| 347 | SCPOLICETROB | Has your use of synthetic weed/cannabis caused you to get in trouble with the police? | 0=No  1=Yes  97=Don’t Know  98=Refused to Answer | If 0 (No) Skip to |
| 348 | SCPROBOTHER | Has your use of synthetic weed/cannabis caused you other problems? | 0=No  1=Yes  97=Don’t Know  98=Refused to Answer |  |
| 349 | SCPROBOTHERO | What other problems has your use of synthetic weed/cannabis caused you? | Alpha Numeric Text Field |  |
| 350 | SCALOT | During the past 12 months, was there a month or more when you spent a lot of your time getting or using synthetic weed/cannabis? | 0=No  1=Yes  97=Don’t Know  98=Refused to Answer |  |
| 351 | SCGETOVER | During the past 12 months, was there a month or more when you spent a lot of your time getting over the effects of the synthetic weed/cannabis you used? | 0=No  1=Yes  97=Don’t Know  98=Refused to Answer |  |
| 352 | SCEFFECTS | During the past 12 months, did you need to use more synthetic weed/cannabis than you used to in order to get the effect you wanted? | 0=No  1=Yes  97=Don’t Know  98=Refused to Answer |  |
| 353 | SCCTDWN | During the past 12 months, did you want to or try to cut down or stop using synthetic weed/cannabis? | 0=No  1=Yes  97=Don’t Know  98=Refused to Answer | If 0(No) skip to |
| 354 | SCCTDWNALO | During the past 12 months, did you cut down or stop using synthetic weed/cannabis at least one time? | 0=No  1=Yes  97=Don’t Know  98=Refused to Answer | If 0(No) skip to |
| 355 | SCSYMPTOMS | Please look at these symptoms. During the past 12 months, did you have any of these symptoms after you cut back or stopped using synthetic weed/cannabis?  *Feeling tired or exhausted  *Having bad dreams  *Having trouble sleeping or sleeping more than usual  *Feeling blue or depressed  *Feeling hungry more often  *Feeling either very slowed down or like you couldn’t sit still | 0=No  1=Yes  97=Don’t Know  98=Refused to Answer |  |
| 356 | SCWORSE | During the past 12 months, did you have any problems with your emotions, nerves, or mental health that were caused or made worse by using synthetic weed/cannabis? | 0=No  1=Yes  97=Don’t Know  98=Refused to Answer | If 0(No) skip to |
| 357 | SCUSEANYWAY | Did you continue to use synthetic weed/cannabis even though you thought it was causing you to have problems with your emotions, nerves, or mental health? | 0=No  1=Yes  97=Don’t Know  98=Refused to Answer |  |
| 358 | SCHLTSPROB | During the past 12 months, did you have any health problems that were probably caused or made worse by your use of synthetic weed/cannabis? | 0=No  1=Yes  97=Don’t Know  98=Refused to Answer | If 0(No) skip to |
| 359 | SCHLTHPROBA | Did you continue to use synthetic weed/cannabis even though you thought it was causing you to have physical health problems? | 0=No  1=Yes  97=Don’t Know  98=Refused to Answer |  |
| 360 | SCPROBFAM | During the past 12 months, did you have any problems with family or friends that were probably caused by your use of synthetic weed/cannabis? | 0=No  1=Yes  97=Don’t Know  98=Refused to Answer | If 0(No) skip to |
| 361 | SCPROBFAMA | Did you continue to use synthetic weed/cannabis even though you thought it caused problems with family or friends? | 0=No  1=Yes  97=Don’t Know  98=Refused to Answer |  |
| 362 | SCFIGHT | Have you ever gotten into a fight or done anything violent after using synthetic weed/cannabis? | 0=No  1=Yes  97=Don’t Know  98=Refused to Answer |  |
| 363 | SCVICTIM | Have you ever been a victim of violence while using synthetic weed/cannabis? | 0=No  1=Yes  97=Don’t Know  98=Refused to Answer |  |
| 364 | SCVIOLENCE | Have you ever seen anyone become violent while using synthetic weed/cannabis? | 0=No  1=Yes  97=Don’t Know  98=Refused to Answer |  |
| 365 | SCER | Have you ever gone to an emergency room after using synthetic weed/cannabis? | 0=No  1=Yes  97=Don’t Know  98=Refused to Answer |  |
| 366 | SCPOISONC | Have you ever called a poison control center after using synthetic weed/cannabis? | 0=No  1=Yes  97=Don’t Know  98=Refused to Answer |  |
| 367 | SCOBTAIN | Now think about the last time you used synthetic weed/cannabis. How did you get this synthetic weed/weed/cannabis? | 1 = Bought it  2 = I traded something else for it  3 = I got it for free or shared someone else’s  4 = I made it myself  97 = Don’t know  98 = Refused to answer |  |
| 367b | SCOBTAINOTH | What other way did you get synthetic weed/cannabis? | Text Field |  |
| 368 | SCFORM | What form of synthetic weed/cannabis did you acquire the last time you used synthetic weed/cannabis? | 1 = Herbal Mixture  2 = Liquid  3 = Other  97 = Don’t Know  98 = Refused to answer | If NE 6 (Other) Skip to 370 |
| 369 | SCFORMOTH | What “other” form of synthetic weed/cannabis did you use? | Alpha Numeric Text Field |  |
| 370 | SCUNIT | What was the unit type of synthetic weed/cannabis that you received the last time you used synthetic weed/cannabis? | 1 = Small bag or sack  2 = Vials/small jars or bottles  3 = Grams  4 = Ounces  5 = Other  97 = Don’t Know  98 = Refused to answer | If NE 5 (Other) Skip to |
| 371 | SCUNITOTHER | What other unit type did you receive? | Alpha Numeric Text Field |  |
| 372 | SCGPRICE | In general, what does synthetic weed/cannabis cost? [Price in dollars] | F5.2 |  |
| 373 | SCGPRICEUNIT | What is the unit of the general price for synthetic weed/cannabis? | 1 = Small bag or sack  2 = Vials/small jars or bottles  3 = Grams  4 = Ounces  5 = Other  97 = Don’t Know  98 = Refused to answer | If NE 3 (Grams) or 4 (Ounces) Skip to |
| 373b | SCGUNITOTHER | What is the "other" unit type? | Text Field |  |
| 374 | SCGPRICEAMT | How many grams/ounces was the general price for synthetic weed/cannabis? | F5.2 |  |
| 375 | SCBUY | Have you ever bought synthetic weed/cannabis yourself? | 0 = No  1 = Yes  97 = Don’t know  98 = Refused to answer | If NE 1(Yes), Skip to |
| 376 | SCLTWHO | The last time you bought synthetic weed/cannabis, who did you buy it from? | 1 = A friend/acquaintance  2 = A relative or family member  3 = Someone I had just met or didn’t know well  4 = The Internet  5 = Delivery service  6 = Store, bodega or smoke shop  7 = Dealer  8 = Other  97 = Don’t know  98 = Refused to answer | If NE 8 (Other) Skip to |
| 377 | SCLTWHOO | What “other” source did you buy synthetic weed/cannabis from? | Alpha Numeric Text Field |  |
| 378 | SCLTBUYWHERE | The last time you bought synthetic weed/cannabis, where were you when you bought it? | 1 = At a club, bar or festival  2 = At a school  3 = At a home, apartment, or dorm  4 = Outside in a public area (e.g., street or park)  5 = Other  97 = Don’t know  98 = Refused to answer | If NE 6 (Other) Skip to |
| 379 | SCLTBUYWHEREO | What “other” location were you in the last time you bought synthetic weed/cannabis? | Alpha Numeric Text Field |  |
| 380 | SCLTBUYRELATION | What is your relationship with your usual synthetic weed/cannabis source? | 1 = Relative  2 = Partner or lover  3 = Work associate  4 = Friend/Acquaintance  5 = Dealer (who is not a friend/relative/partner)  6 = No personal relationship  97 = Don’t know  98 = Refused to answer |  |
| 381 | SCLTBUYUNIT | The last time you bought synthetic weed/cannabis, what units did you buy it in? | 1 = grams  2 = ounces  3 = bags  4 = vials/small jars or bottles  5 = other  97 = Don’t know  98 = Refused to answer | Else if NE 5 (Other) Skip to |
| 382 | SCLTBUYUNITO | The last time you bought synthetic weed/cannabis what “other” unit did you buy it in? | Alpha Numeric Text Field |  |
| 383 | SCLTBUYAMT | The last time you bought synthetic weed/cannabis, what number of gms/ozs/bags/vials/other [from item 145] did you buy | F5.2 |  |
| 384 | SCLTMONEY | The last time you bought synthetic weed/cannabis, how much money did you spend? (whole dollars) | F4.0 |  |
| 385 | SCLTMONEYW | How did you get the money that you spent the last time you bought synthetic weed/cannabis? | 1 = Legal employment  2 = Public assistance  3 = Partner or spouse  4 = Parents  5 = Other family member  6 = Drug sales  7 = Sex work  8 = Oher illegal hustles  9 = Legal hustles or off-book work  10 = Savings  11 = Shelter or relief funds  13 = Other  97 = Don’t know  98 = Refused to answer | If NE 13 (Other) skip to |
| 386 | SCLTMONEYWO | What “other” source did you use to get the money that you spent the last time you bought synthetic weed/cannabis? | Alpha Numeric Text Field |  |
| 387 | SCLTWHAT | The last time you bought synthetic weed/cannabis, what were you buying? | 1 = Fubinaca  2 = Pinaca  3 = Chiminaca  4 = JWH-xxx  5 = Other  97 = Don’t know  98 = Refused to answer | If NE 5 (Other) Skip to |
| 388 | SCLTWHATO | What “other” were you buying the last time you bought synthetic weed/cannabis? | Alpha Numeric Text Field |  |
| 389 | SCLTEXP | Was the quality or experience what you expected the last time you used synthetic weed/cannabis? | 0=No  1=Yes  97 = Don’t know  98 = Refused to answer | If NE 1(Yes) Skip to |
| 390 | SCLTEXPDIFF | The last time you used synthetic weed/cannabis how was the quality or experience different from your expectations? | 1 = Too stimulating (nervousness, anxiety, anger)  2 = Too calming (fatigue, lethargy)  3 = Other  97 = Don’t know  98 = Refused to answer | If NE 3(Other) Skip to |
| 391 | SCLTEXPDIFFO | The last time you used synthetic weed/cannabis, what was the “other” way your experience was different from your expectations? | Alpha Numeric Text Field |  |
| 392 | OSYNTHETICS | Have you ever used any other synthetic drugs? | 0 = No  1 = Yes  97 = Don’t Know  98 = Refused to Answer | If 0 (No) Skip to |
| 393 | OSYNTHETICSWHAT | What other synthetic drug have you used? | Alpha numeric text field |  |
| 394 | OSLAST | How long has it been since you last used [named synthetic]? | 1 =- Within the past 30 days  2 = More than 30 days ago but within the past 12 months  3 = More than 12 months ago  97 = Don’t Know  98 = Refused to Answer | If 3 (More than 12 months ago) Skip to |
| 395 | OSDAYS | On average, how many days did you use [named synthetic] each month during the past 12 months? | F2.0 |  |
| 396 | OS30DAYS | Think specifically about the past 30 days up to and including today. On how many of those days did you use [named synthetic]? | F2.0 |  |
| 397 | INJDRUGS | Have you ever in your life shot up or injected any drugs other than those prescribed for you? By shooting up I mean anytime you might have used drugs with a needle, either by mainlining, skin popping, or muscling? | 0=No  1=Yes  97=Don’t Know  98=Refused to Answer | If 0(No) Skip to |
| When you last injected drugs, did you inject… | | | | |
| 398 | INJECTBS | Did you inject bath salts? | 0=No  1=Yes  97=Don’t Know  98=Refused to Answer |  |
| 399 | INJECTMO | Did you inject Molly? | 0=No  1=Yes  97=Don’t Know  98=Refused to Answer |  |
| 400 | INJECTHN | Did you inject heroin? | 0=No  1=Yes  97=Don’t Know  98=Refused to Answer |  |
| 401 | INJECTMT | Did you inject Methamphetamine? | 0=No  1=Yes  97=Don’t Know  98=Refused to Answer |  |
| 402 | INJECTCR | Did you inject crack? | 0=No  1=Yes  97=Don’t Know  98=Refused to Answer |  |
| 403 | INJECTOTHER | Were there other drugs you injected? | 0=No  1=Yes  97=Don’t Know  98=Refused to Answer | If 0(N0) Skip to |
| 404 | INJECTOTHERO | What other drugs did you inject? | Alpha Numeric Text Field |  |
| 405 | INJDRUGSWHN | How long has it been since you injected any drug? | 1 =- Within the past 30 days  2 = More than 30 days ago but within the past 12 months  3 = More than 12 months ago  97 = Don’t know  98 = Refused to answer | If 3 (More than 12 months ago) Skip to |
| 406 | INJSTERILE | In the past 12 months, when you injected, how often did you use a new, sterile needle? By a new, sterile needle, I mean a needle that has never been used before by anyone, even you? | 1 = Never  2 = Rarely  3 = About half the time  4 = Most of the time  5 = Always  97 = Don’t know  98 = Refused to answer | If 5(Always), Skip to |
| 407 | INJALRINJ | In the past 12 months, how often did you use needles that someone else had already injected with? | 1 = Never  2 = Rarely  3 = About half the time  4 = Most of the time  5 = Always  97 = Don’t know  98 = Refused to answer | If 1 (Never) skip to |
| 408 | INJAFTPEOPLE | In the past 12 months, with how many people did you use a needle after they injected with it? | F3.0 |  |
| 409 | INJCCWUSED | In the past 12 months, how often did you use cookers, cottons, or water that someone else had already used? | 1 = Never  2 = Rarely  3 = About half the time  4 = Most of the time  5 = Always  97 = Don’t know  98 = Refused to answer | If 1(Never) Skip to |
| 410 | INJCCW | In the past 12 months, with how many people did you use the same cooker, cotton, or water that they had already used? By “water” I mean for rinsing needles or preparing drugs. | F3.0 |  |
| 411 | INJSYRUSED | In the past 12 months when you injected, how often did you use drugs that had been divided with a syringe that someone else had already injected with? | 1 = Never  2 = Rarely  3 = About half the time  4 = Most of the time  5 = Always  97 = Don’t know  98 = Refused to answer | If 1(Never) Skip to |
| 412 | INJUSEDSER | In the past 12 months, with how many people did you use drugs that had been divided with a syringe that had already been used? | F3.0 |  |
| 413 | SEXWOMEN | In the past six months, how many women have you had sex with, including vaginal, anal, oral? | F3.0 | If 0(Zero) Skip to |
| 414 | SEXWOMENUP | With how many of these female sex partners did you not use condoms or other protection? | F3.0 |  |
| 415 | SEXWOMENYPAY | How many of these female sex partners did you give drugs or money to in exchange for sex? | F3.0 |  |
| 416 | SEXWOMENTPAY | How many of these female sex partners did you provide sex to in exchange for drugs or money? | F3.0 |  |
| 417 | SEXMEN | In the past 6 months, how many men have you had sex with, including ~~vaginal~~, anal, oral? | F3.0 | If 0(Zero) Skip to |
| 418 | SEXMENUP | With how many of these male sex partners did you not use condoms or other protection? | F3.0 |  |
| 419 | SEXMENYPAY | How many of these male sex partners did you give drugs or money to in exchange for sex? | F3.0 |  |
| 420 | SEXMENTPAY | How many of these male sex partners did you provide sex to in exchange for drugs or money? | F3.0 |  |
| The next few questions ask about violence: physical fights, cutting, beatings, violent language and/or family disturbances. | | | | |
| 421 | WEAPONDAYS | During the past 30 days, on how many days did you carry a weapon such as a gun, knife, or club? | 1 = 0 days  2 = 1 day  3 = 2 or 3 days  4 = 4 or 5 days  5 = 6 or more days  97 = Don’t know  98 = Refused to answer | If 0(Zero) Skip to |
| 422 | GUNDAYS | During the past 30 days, on how many days did you carry a gun? | 1 = 0 days  2 = 1 day  3 = 2 or 3 days  4 = 4 or 5 days  5 = 6 or more days  97 = Don’t know  98 = Refused to answer |  |
| 423 | THREATENED | During the past 12 months, how many times has someone threatened or injured you with a weapon such as a gun, knife, or club? | 1 = 0 times  2 = 1 time  3 = 2 or 3 times  4 = 4 or 5 times  5 = 6 or 7 times  6 = 8 or 9 times  7 = 10 or 11 times  8 = 12 or more times  97 = Don’t know  98 = Refused to answer |  |
| 424 | FIGHT | During the past 12 months, how many times were you in a physical fight? | 1 = 0 times  2 = 1 time  3 = 2 or 3 times  4 = 4 or 5 times  5 = 6 or 7 times  6 = 8 or 9 times  7 = 10 or 11 times  8 = 12 or more times  97 = Don’t know  98 = Refused to answer |  |
| 425 | BFGFHURT | During the past 12 months, did your partner, spouse, boyfriend or girlfriend ever hit, slap, or physically hurt you? | 0=No  1=Yes  97=Don’t Know  98=Refused to Answer |  |
| 426 | SEXFORCED | Have you ever been forced to have sex when you did not want to? | 0=No  1=Yes  97=Don’t Know  98=Refused to Answer |  |
| I am now going to ask you questions about sex and sexual health. These questions are personal, so please remember that this survey is confidential. | | | | |
| 427 | HIVTESTE | Have you ever been tested for HIV? | 0=No  1=Yes  97=Don’t Know  98=Refused to Answer | If 0(No) Skip to |
| 428 | HIVTEST2YR | In the past 2 years, how many times have you been tested for HIV? | F3.0 |  |
| 429 | HIVTEST2YRR | When you were tested for HIV in the past 2 years, did you get the result of the test? | 0=No  1=Yes  97=Don’t Know  98=Refused to Answer | If 1(Yes) Skip to |
| 430 | HIVTESTNOW | What was the main reason you did not get your HIV test result? | Alpha Numeric Text Field |  |
| 431 | HIVTESTRES | What was the result of your most recent HIV test? | 1 = Negative  2 = Positive  3 = Indeterminate  97 = Don’t know  98 = Refused to answer |  |
| 432 | HERPES | Have you ever been diagnosed with herpes? | 0=No  1=Yes  97=Don’t Know  98=Refused to Answer |  |
| 433 | CHLAMYDIA | Have you ever had Chlamydia? | 0=No  1=Yes  97=Don’t Know  98=Refused to Answer |  |
| 434 | GONORRHEA | Have you ever had gonorrhea? | 0=No  1=Yes  97=Don’t Know  98=Refused to Answer |  |
| 435 | SYPHILIS | Have you ever had syphilis? | 0=No  1=Yes  97=Don’t Know  98=Refused to Answer |  |
| 436 | HPV | Have you ever been diagnosed with Human Papillomavirus (HPV) | 0=No  1=Yes  97=Don’t Know  98=Refused to Answer |  |
| 437 | TRICHOMONIASIS | Have you ever had Trichomoniasis? | 0=No  1=Yes  97=Don’t Know  98=Refused to Answer |  |
| The rest of our questions pertain to your overall health. | | | | |
|  | HCV | Have you ever been diagnosed with Hepatitis C (HCV)? | 0=No  1=Yes  97=Don’t Know  98=Refused to Answer |  |
| 4438 | HLTHDRUGP | Do you have any health problems now that you think are related to drug use? | 0=No  1=Yes  97=Don’t Know  98=Refused to Answer | If 0(No) end of survey |
| 439 | HLTHDRUGPS | What health problems do you think are related to drug use? | Alpha Numeric Text Field |  |
|  |  |  |  |  |
